# Dynamic Mirror-Symmetry Breaking in Bicontinuous Cubic Phases**

**DOI:** 10.1002/anie.201406907

**Published:** 2014-09-26

**Authors:** Christian Dressel, Feng Liu, Marko Prehm, Xiangbing Zeng, Goran Ungar, Carsten Tschierske

**Affiliations:** Institute of Chemistry, Organic Chemistry, Martin-Luther-University Halle-WittenbergKurt-Mothes-Str. 2, 06120 Halle (Germany); Department of Engineering Materials, University of Sheffield, Robert Hadfield BuildingMappin Street, Sheffield S1 3JD (UK); State Key Laboratory for Mechanical Behavior of Materials, Xi'an Jiaotong UniversityXi'an 710049 (P.R. China); Department of Physics, Zhejiang Sci-Tech University, Xiasha College ParkHangzhou 310018 (China)

**Keywords:** chiral isotropic liquid, conglomerate, deracemization, polycatenar liquid crystal, spontaneous chiral induction

## Abstract

Chiral segregation of enantiomers or chiral conformers of achiral molecules during self-assembly in well-ordered crystalline superstructures has fascinated chemists since Pasteur. Here we report spontaneous mirror-symmetry breaking in cubic phases formed by achiral multichain-terminated diphenyl-2,2′-bithiophenes. It was found that stochastic symmetry breaking is a general phenomenon observed in bicontinuous cubic liquid crystal phases of achiral rod-like compounds. In all compounds studied the ${{\it Im}\bar 3m}$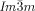 cubic phase is always chiral, while the ${Ia\bar 3d}$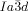 phase is achiral. These intriguing observations are explained by propagation of homochiral helical twist across the entire networks through helix matching at network junctions. In the ${Ia\bar 3d}$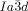 phase the opposing chiralities of the two networks cancel, but not so in the three-networks ${{\it Im}\bar 3m}$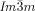 phase. The high twist in the ${{\it Im}\bar 3m}$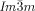 phase explains its previously unrecognized chirality, as well as the origin of this complex structure and the transitions between the different cubic phases.

Design and investigation of chiral self-assembled superstructures represents a fascinating field of contemporary research which provides numerous potential applications. Beside enantiomeric mixtures of molecules, segregation of chiral supermolecular aggregates,[1,2,3] and chiral molecular conformations were also found as sources of macroscopic chirality in the crystalline state and at surfaces.[4] In recent years interest in mirror-symmetry breaking extended to soft matter systems. Special progress in this field was made with liquid crystalline phases formed by bent-core molecules and bent-shaped mesogenic dimers showing symmetry breaking in their lamellar[5–11] and nematic phases, respectively.[12,13] Spontaneous formation of helical superstructures was also observed in soft columnar crystals at reduced temperature where packing density is increased and the mobility of the aromatic segments is reduced.[14–17] The lateral coupling between columns is much weaker than the longitudinal, therefore macroscopic chiral segregation is usually not observed, except for one case.[10] Though, there is one report about spontaneous stochastic symmetry breaking in a thermotropic cubic phase, the structure of this cubic phase and the possible origin of macroscopic chirality are not clear in this case.[18]

Herein we report that stochastic symmetry breaking is a general phenomenon typically observed in the bicontinuous cubic phases of achiral rod-like and polycatenar (multichain-terminated) liquid crystalline (LC) compounds.[19] We show that optical activity and circular dichroism (CD) develop spontaneously whenever the cubic phase is of the triple-network type with ${{\it Im}\bar 3m}$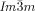 symmetry[20,21] whereas, in contrast, the “double gyroid” ${Ia\bar 3d}$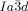 cubic phase always remains achiral. These observations are explained by the action of two newly recognized phenomena: a) the correlated helical twist of molecular axis in the segments of the infinite interpenetrating networks, and b) the percolation through mm-sized domains of uniform helical sense carried across the network through matching of molecular orientation twist sense at network junctions. The current findings also bring the understanding of thermotropic cubic phases to a qualitatively new level and contribute to the general appreciation of symmetry breaking in LCs of achiral molecules.

The compounds under investigation (compounds **1**, see Table 1) represent polycatenar rod-like molecules based on a 5,5′-diphenylbithiophene core (for synthesis, see the Supporting Information (SI)).[23] Crystalline phases of compounds **1** melt between 110 and 139 °C and form optically isotropic and highly viscous mesophases which, based on X-ray diffraction (XRD) evidence (see below), are cubic LC phases, and which on further rising temperature transform to isotropic liquids. In some cases, an additional liquid-liquid transition (LLT) is observed and the liquid phase occurring between the cubic and the “ordinary” isotropic liquid is labelled here Iso_LT,_ the liquid phase at higher temperature as Iso_HT_. Iso_LT_ phases have previously been found as intermediate phases at Cub-Iso transitions (labelled Iso_1_)[22] and have recently been found to be chiral in some cases, representing a conglomerate of two segregated enantiomeric liquids (Iso_1_^[*]^).[23] This chiral Iso_LT_ is denoted here as Iso_LT_^[*]^. While Iso_LT_^[*]^ appears in **1 d** both on heating and cooling,[23] in **1 b** and **1 e** it occurs only on cooling (monotropic phase). Compounds **1 a, 1 c, 1 f** and **1 g** form the cubic phases directly from the achiral Iso_HT_. Sharp DSC peaks accompany Cub-Iso transitions on heating and Iso_HT_-Cub transitions on cooling, but the Iso_HT_-Iso_LT_^[*]^ exotherm is broad and is better described as a heat capacity anomaly rather than a first-order transition (see Figure 3 c,d, Table 1 and Figures S1–S9 in the SI).

**Table 1:** Chemical structures, phase transitions (heating top lines, cooling bottom lines) and cubic lattice parameters of the compounds of series 1.[a] 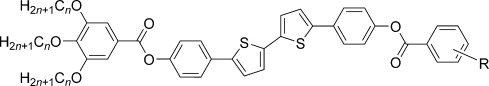

Compd.	*n*	R	Phase transitions on heating/cooling (*T*/°C)	*a*_cub_ [nm]
**1 a**	10	H	Cr 114 [39.7] Cub/${Ia\bar 3d}$ 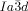 162 [2.3] Iso_HT_ Iso_HT_ 156 [1.7] Cub/${Ia\bar 3d}$ 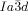 69 [3.8] Cr	10.8
**1 b**	10	4-OCH_3_	Cr 119 [55.3] Cub/${Ia\bar 3d}$ 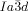 192 [1.5] Iso_HT_ Iso_HT_ 192 [0.2] Iso_LT_^[^*^]^ 170 [0.3] Cub/${Ia\bar 3d}$ 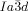	11.4
**1 c**	10	3,4-(OCH_3_)_2_	Cr 137 [44.6] Cub/${Ia\bar 3d}$ 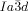 183 [3.3] Iso_HT_ Iso_HT_ 175 [2.5] Cub/${Ia\bar 3d}$ 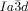 105 [37.3] Cr	11.1
**1 d**[23]	6	4-OC_6_H_13_	Cr 139 [55.2] M 171 [-] Cub/${Ia\bar 3d}$ 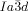 205 [1.2] Iso_LT_^[^*^]^ 213 [0.2] Iso_HT_ Iso_HT_ 212 [1.0] Iso_LT_^[^*^]^ 177 [0.3] Cub/${Ia\bar 3d}$ 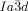 58 [19.4] Cr	10.3
**1 e**	10	4-OC_10_H_21_	Cr 134 [49.9] Cub^[^*^]^/${{\it Im}\bar 3m}$ 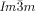 183 [2.2] Iso_HT_ Iso_HT_ 178 [0.3] Iso_LT_^[^*^]^ 173 [1.1] Cub^[^*^]^/${{\it Im}\bar 3m}$ 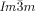 109 [46.0] Cr	17.9
**1 f**	10	3-OC_2_H_5_	Cr 100 [27.8] Cub^[^*^]^/${{\it Im}\bar 3m}$ 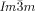 130 [3.1] Iso_HT_ Iso_HT_ 121 [2.1] Cub^[^*^]^/${{\it Im}\bar 3m}$ 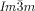 <20 Cr	15.7
**1 g**	10	3,4,5-(OCH_3_)_3_	Cr 110 [70.2] Cub^[^*^]^/${{\it Im}\bar 3m}$ 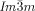 127 [2.9] Iso_HT_ Iso_HT_ 117 [2.1] Cub^[^*^]^/${{\it Im}\bar 3m}$ 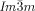	15.4

[a] Abbreviations: Cr=crystalline solid, Cub/${Ia\bar 3d}$
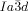
=bicontinuous cubic phase with ${Ia\bar 3d}$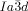 lattice (achiral); Cub^[^*^]^/${{\it Im}\bar 3m}$
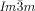
=chiral conglomerate cubic phase with ${{\it Im}\bar 3m}$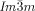 lattice; Iso_LT_^[^*^]^=chiral isotropic liquid phase formed by a conglomerate of domains with opposite handedness; Iso_HT_=achiral isotropic liquid phase.

Cubic space groups were determined by powder small-angle XRD and grazing-incidence diffraction as shown in the SI (Figures S11, S12, and Tables S2–S8). Two cubic phases were observed, the double-network “gyroid”, with ${Ia\bar 3d}$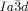 symmetry (Figure 1 a), and the triple-network phase with ${{\it Im}\bar 3m}$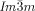 symmetry (Figure 1 b). The lattice parameters of the cubic phases are in the typical ranges around *a*_cub_=11 nm for the ${Ia\bar 3d}$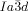 phase and *a*_cub_=15–18 nm for the ${{\it Im}\bar 3m}$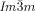 phase.

**Figure 1:**
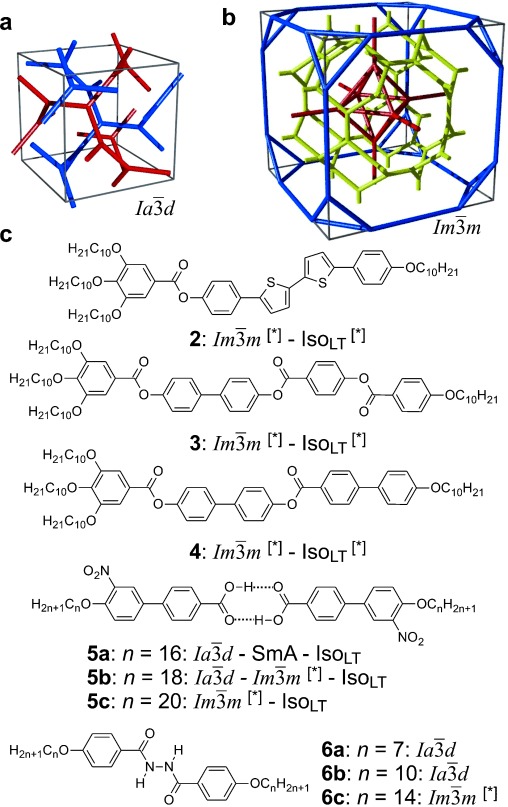
a,b) Framework models of a unit cell of the two cubic structures discussed in this work[20] (see also Figures S15 and S16) and c) typical chemical structures of representative compounds forming these phases (compounds 2–4 were newly synthesized, as described in the SI, and compounds 5 and 6 were known from literature, see Table S1 and Figures S7–S9, S13, S14, S17–S22 for details.[19] a) The double-network ${Ia\bar 3d}$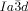 (“gyroid”) phase; b) the triple-network ${{\it Im}\bar 3m}$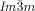 phase. Each of the infinite networks is coloured differently. In (b) the red and blue are the identical “inner” and “outer” networks, mutually related by a (1/2 1/2 1/2) translation; yellow is the “middle” network. Equivalent figures with the added minimum surface are shown in SI.

Symmetry breaking is observed in some of the cubic phases by polarized microscopy if the analyzer is rotated by a small angle out of the 90 °C crossed position with respect to the polarizer. In this way the polarizing microscope can be used as a polarimeter with the additional advantage of spatial resolution. Remarkably, all ${Ia\bar 3d}$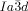 cubic phases of the investigated compounds are optically inactive, whereas for all ${{\it Im}\bar 3m}$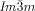 phases darker and brighter domains become visible between slightly uncrossed polarizers, which exchange their contrast if the rotation of the analyzer is reversed (Figure 2 a,b). Rotating the sample between the polarizers does not change the contrast. This means that the ${{\it Im}\bar 3m}$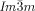 phase represents a conglomerate of chiral domains with opposite handedness. Thus this cubic phase is denoted Cub^[*]^/${{\it Im}\bar 3m}$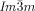.[24]

**Figure 2:**
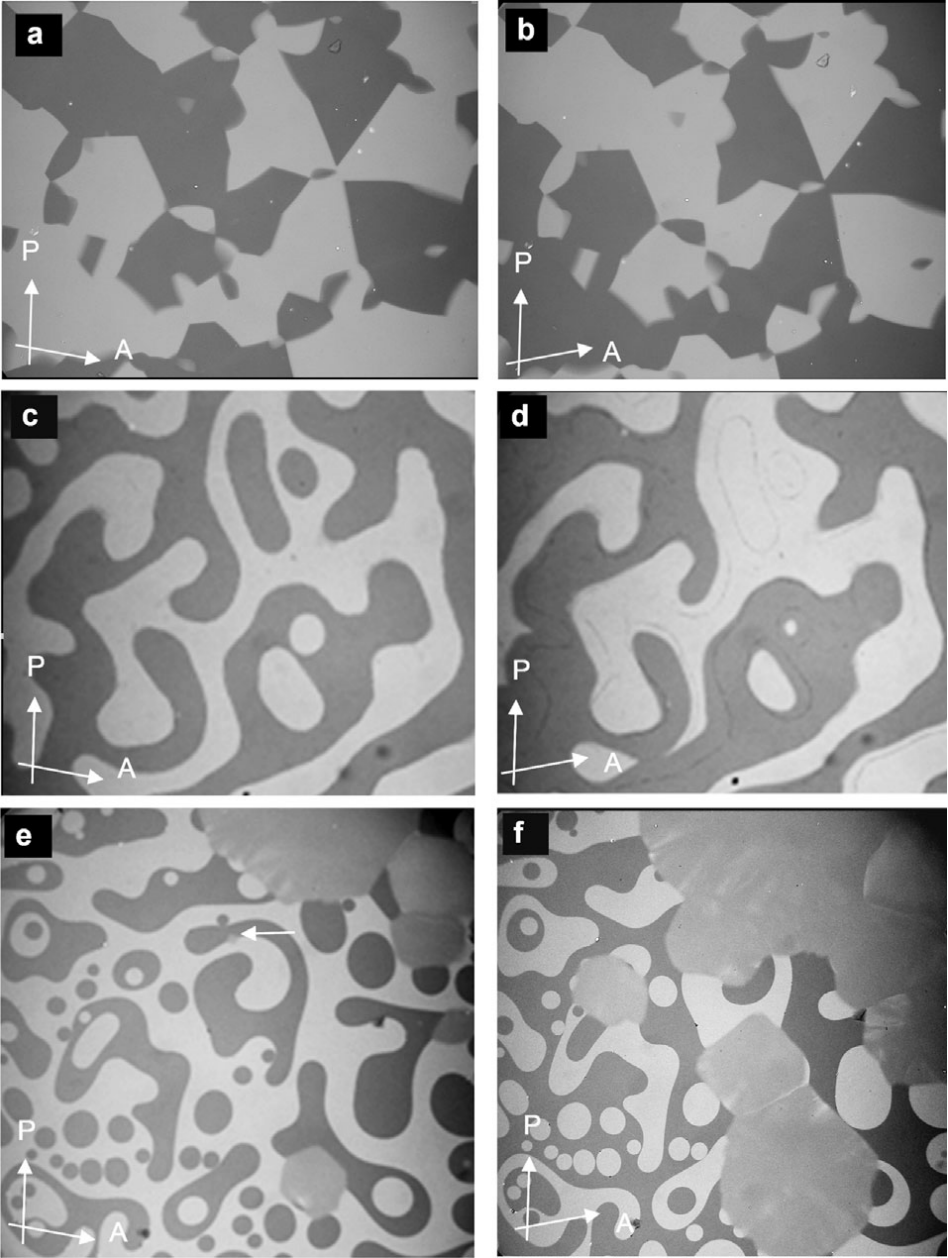
Photomicrographs of chiral domains (dark/bright), observed between slightly uncrossed polarizers. The orientations of polarizer (P) and analyzer (A) are indicated by arrows. a,b) ${{\it Im}\bar 3m}$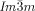 phase of compound 1 f at *T*=112 °C as obtained on cooling from the achiral Iso_HT_ phase, c) Iso_LT_^[*]^ phase of compound 1 e (*T*=177 °C) and d) ${{\it Im}\bar 3m}$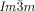 phase (*T*=175 °C) as observed after transition from the Iso_LT_^[*]^ phase; note that the domain boundaries between the chiral domains are slightly shifted. e,f) Growths of the domains of the ${Ia\bar 3d}$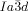 phase at the Iso_LT_^[*]^-${Ia\bar 3d}$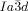 transition as observed for compound 1 b at *T*=160 °C (white arrow indicates a seed of the ${Ia\bar 3d}$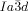 phase); note that during formation of the cubic phase the chirality of the Iso_LT_^[*]^ phase is completely extinguished (see also videos in SI).

Microbeam circular dichroism (CD) spectroscopy confirmed the presence of chiral domains in the ${{\it Im}\bar 3m}$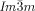 phase. Figure 3 a shows the CD evolution with temperature during heating a bulk film of **1 g** from the Cub^[*]^/${{\it Im}\bar 3m}$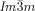 (120–125 °C) to the Iso_HT_ phase (130–140 °C), and then cooling back to the Cub^[*]^/${{\it Im}\bar 3m}$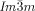 (120–125 °C, dashed curves). As can be seen, there is strong CD in the cubic phase, disappearing in the isotropic Iso_HT_ liquid and reappearing in the Cub^[*]^/${{\it Im}\bar 3m}$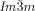, this time either with the same or with reversed chirality. In contrast, on no occasion did the Cub/${Ia\bar 3d}$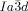 phase give any measurable CD.

**Figure 3:**
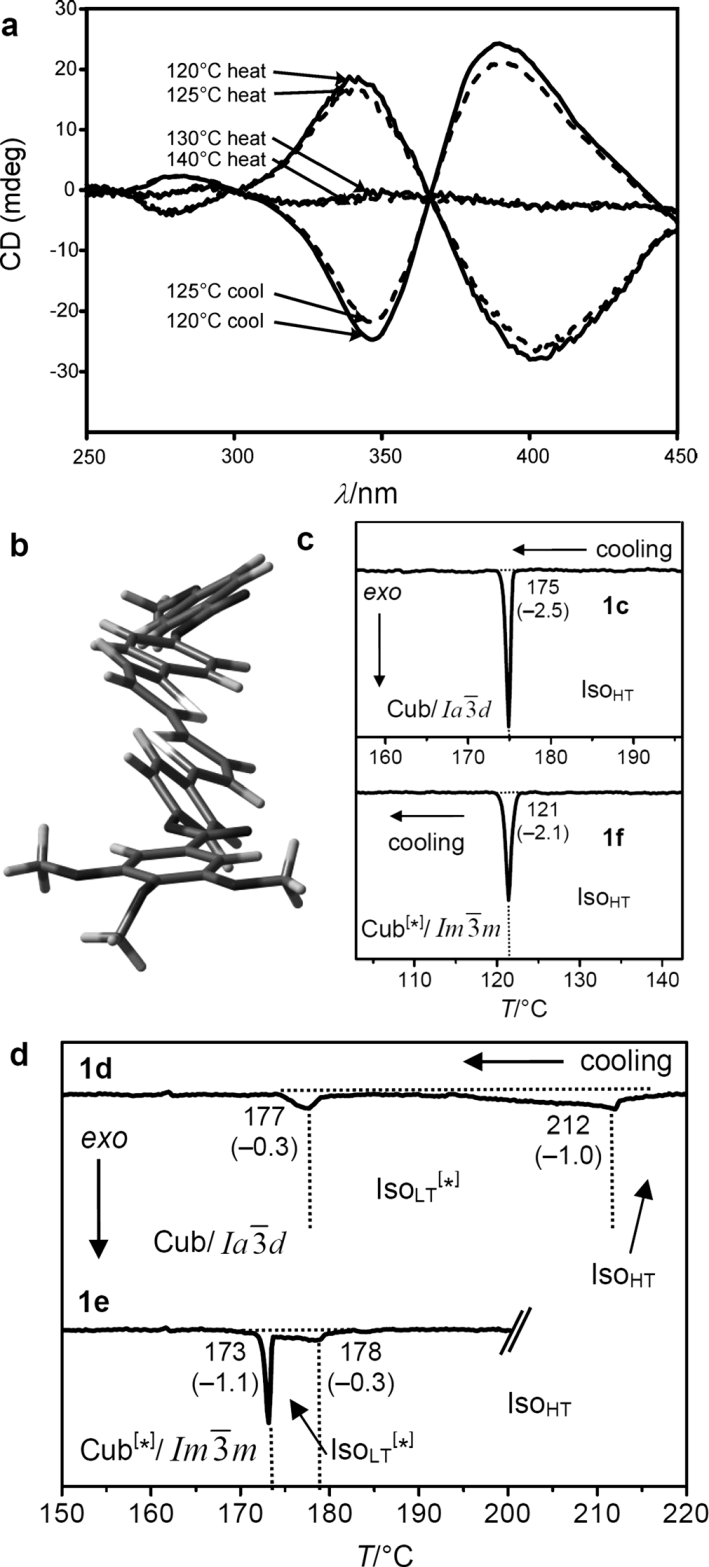
a) Temperature dependent CD spectra (ellipticity in mdeg) of a bulk film of compound 1 g in the Cub^[*]^/${{\it Im}\bar 3m}$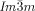 (120–125 °C) and the Iso_HT_ liquid (130–140 °C). On cooling back to the Cub^[*]^/${{\it Im}\bar 3m}$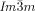 (dashed curves) chirality reverses. b) Helical conformations as computed for a model compound related to compounds 1 b,e with OCH_3_ groups instead of the long alkyloxy chains.[23] c,d) DSC cooling thermograms of c) 1 c and 1 f with direct Iso_HT_-Cub transitions and d) 1 d[23] and 1 e with an intermediate Iso_LT_^[*]^ phase (see also Figures S1–S6).

While both, optical activity and CD, are strongly affected by helical order, CD is highly sensitive to molecular conformation. It is suggested that the chiral chromophore mainly responsible for the CD is the slightly twisted 5,5′-diphenyl-2,2′-bithiophene unit (Figure 3 b) which has its UV absorption maximum at the inflection point of the CD curves at 374 nm (Figure S10).

As mentioned above, there are two different types of transitions from the achiral Iso_HT_ liquid to the Cub^[*]^/${{\it Im}\bar 3m}$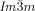 phase, either directly (compounds **1 f** and **1 g**, Figure 2 a,b) or via the chiral Iso_LT_^[*]^ phase (compounds **1 e** and **2**, Figure 2 c,d). The chirality of the Iso_LT_^[*]^ phase, a conglomerate of chiral domains, is indicated by optical investigations in the same way as described for the cubic phases (see Figure 2 c). Where the transition takes place directly from the achiral Iso_HT_ phase the resulting chiral domains have mainly straight boundaries reflecting crystallographic facets (Figure 2 a,b). However, when the ${{\it Im}\bar 3m}$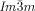 phase grows from the already chiral Iso_LT_^[*]^ phase, the chiral cubic domain boundaries follow closely the curved borders between the enantiomeric liquid domains of the Iso_LT_^[*]^ phase (see Figure 2 c,d and Video 1e on the SI website).

The transition to the ${Ia\bar 3d}$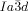 phase can also occur in two ways. Other than by XRD, the direct transition Iso_HT_-Cub/${Ia\bar 3d}$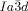 is only detectable by a marked increase in viscosity (compounds **1 a** and **1 c**). In this case investigation between uncrossed polarizers give no evidence of chirality. What is more, where the ${Ia\bar 3d}$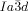 is formed from the chiral Iso_LT_^[*]^ phase (compounds **1 b** and **1 d**), the chirality is completely erased (see Figure 2 e,f).

Similar Iso_LT_^[*]^-Cub^[*]^/${{\it Im}\bar 3m}$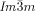 transition behavior as for compound **1 e**, was also observed for compound **2**, having a shorter bithiophene unit and for the tetracatenar compounds **3** and **4** without the bithiophene units. **5** (ANBC-*n*) and **6** (BABH-*n*) are members of the two best investigated homologous series of cubic phase forming compounds (see Table S1 and Figures 1, S7–S9, S13, S14, S17–S22).[19,25] Remarkably, also for these compounds in all cases the ${Ia\bar 3d}$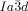 phase is achiral (compounds **5 a, b** and **6 a**, **b**) whereas the ${{\it Im}\bar 3m}$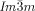 phase appears as chiral conglomerate (compounds **2**–**4**, **5 b**, **c** and **6 c**). For the 3′-nitro-4′-alkoxybiphenyl carboxylic acids **5** there is an additional enantiotropic (i.e. reversible) Iso_HT_–Iso_LT_ transition; however in this case the Iso_LT_ phase is for all investigated compounds achiral. Nevertheless, chirality evolves for **5 c** (*n*=20) at the transition to the Cub^[*]^/${{\it Im}\bar 3m}$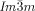 phase (see Table S1 and Figure S20e,f). For **5 b** (*n*=18) an achiral ${Ia\bar 3d}$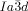 phase is formed on cooling, but on heating an additional ${Ia\bar 3d}$
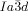
–${{\it Im}\bar 3m}$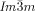 transition occurs at *T*≈180 °C (Figure S20c,d).[19] Again, chiral domains invariably appear at this transition. Thus, the Cub/${{\it Im}\bar 3m}$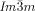 phase was found to be chiral in all these diverse non-chiral polycatenar compounds, without exception,[24] while the Cub/${Ia\bar 3d}$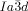 was non-chiral, also without exception. Hence, chirality of the ${{\it Im}\bar 3m}$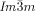 phases appears to be a general phenomenon, already present in long known systems, but surprisingly not previously recognized.[26] Chirality in the Cub^[*]^/${{\it Im}\bar 3m}$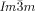 phase has thus been seen to develop in four different ways, either 1) directly from the achiral isotropic liquid Iso_HT_, via Iso_LT_ phases which can be either 2) chiral (Iso_LT_^[*]^) or 3) achiral (Iso_LT_), or 4) from the achiral Cub/${Ia\bar 3d}$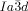 phase.

For the understanding of the development of chirality in the cubic phase it must be recalled that the molecules have liquid like local order, with no hint of any Bragg-like X-ray reflection in the wide-angle range. Therefore, the classical approach of achiral symmetry breaking, where the formation of a more or less ordered crystalline phase energetically favors one chiral conformer over another,[1,3,7] cannot be applied here. Considering that the observed “static symmetry breaking” leads to a significant entropy penalty, there is a compelling mode of self-assembly that compensates for the entropy loss, yet does not involve crystallization at any level (“dynamic symmetry breaking”), as introduced recently as the underlying process for mirror-symmetry breaking in isotropic liquids.[23] The model must also explain why all Cub/${{\it Im}\bar 3m}$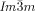 phases are chiral and all Cub/${Ia\bar 3d}$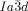 ones are not.

In soft-crystal columns of short hexacatenar hydrazine rods[27] or broader board-like perylene bisimides[28] it was found that molecular pairs stack on top of one another with a nearly 90° twist. In this way the molten alkyls spread around the column while effective core–core π-stacking is still maintained. For long thin rods with less than six chains, as studied here, a smaller twist angle would be sufficient to alleviate the crowding of the alkyls,[19,29] thus allowing the development of helical twist. Without any degree of 3D positional order of achiral molecules, as in the “disordered” hexagonal columnar LC phase, there is no long-range helical order (LRHO). However, the superstructural twist couples with the helical twist of the chiral molecular conformations, thus biasing each other and collectively favoring the helical superstructure in the fluid columns.[23] Thus, the preorganization in columns favors conformational segregation and vica verse. But even in this case, in 1D columns, without intercolumnar correlation, a helix reversal defect has no way of being corrected, so that macroscopic chirality cannot be observed in fluid columnar LC phases.

However, in the bicontinuous cubic phases we also must consider the network junctions. At the 3-fold junctions the three twisting “ribbons” that may be used to represent the network segments, must merge into one another smoothly. In an optimal junction the aromatic rods arrive parallel to each other and with a synchronized twist where the helices lock-in together as they approach each other—for the ${Ia\bar 3d}$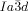 network junction (see Figure 4 b,c). The junction itself then effectively becomes a small triangular platelet, approximately normal to the molecular axis.[19,25] We calculate that there is a distance of 0.345 *a*_cub_≈3.9 nm between the junctions, which accommodates about 9 molecular triplets spaced at 0.45 nm. As the twist between the ${Ia\bar 3d}$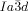 junctions is *ϕ*=±70.5°, there is an average rotation of ±8° between adjacent molecules. For details see SI. As is well known, the two networks in the ${Ia\bar 3d}$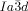 phase have opposite chirality; the blue network in Figures 1 a and 4 a is right-handed and the red network is left-handed. Thus, although there is long-range helical order in each of the two interpenetrating infinite networks of the ${Ia\bar 3d}$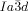 phase, there is no net chirality as the two cancel completely. Figure 4 a gives an impression of the proposed ${Ia\bar 3d}$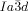 structure, including the minimum surface that separates the two networks (see also SI).

**Figure 4:**
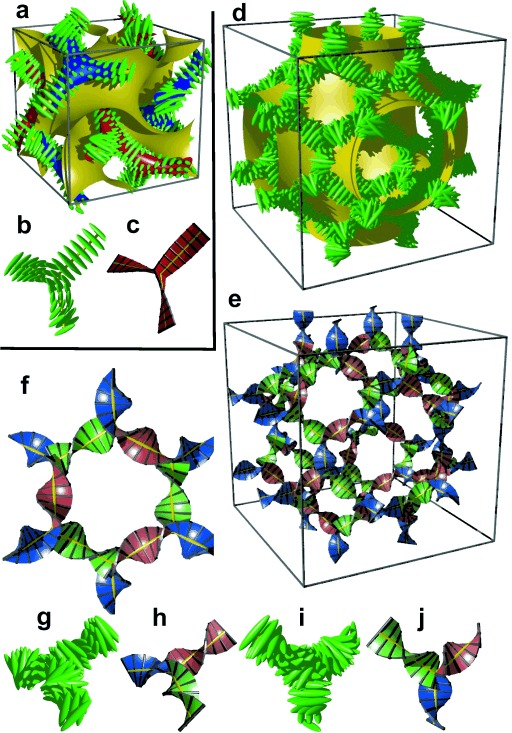
a) The two networks (red and blue) of the ${Ia\bar 3d}$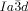 phase decorated with schematic mesogens (rod-like molecular cores, green) showing the molecular twist along the network segments. The gyroid minimum surface is also shown (yellow) and b,c) show the network junctions. d) The same for the middle of the three networks of the ${{\it Im}\bar 3m}$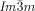 phase (yellow network in Figure 1 b). This network closely follows the Schwartz *P*-type minimum surface (shown in yellow). e) The middle ${{\it Im}\bar 3m}$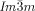 network shown as ribbons containing the molecular axes axis (black rods) and f) loop of 6 junctions in this network. g–j) Details of the two types of junctions in the ${{\it Im}\bar 3m}$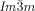 phase in mesogen (g,i) and ribbon (h,j) representations.

Turning to the ${{\it Im}\bar 3m}$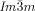 phase, this contains three rather than two networks,[20,21] a fact on which we base our explanation of its chirality. Examination of the three networks in Figure 1 b shows that they are not intrinsically chiral. However, it is reasonable to assume that they also contain molecular twist as in the ${Ia\bar 3d}$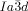 phase. In fact even more so, as the molecules displaying the ${{\it Im}\bar 3m}$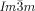 phase (e.g. **1 e**–**1 g**) generally have larger terminal groups than ${Ia\bar 3d}$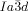-forming compounds (e.g. **1 a**–**1 d**). They are thus expected to cause greater steric crowding. We concentrate here only on the middle (yellow) network, as it is the simplest of the three and, like in the ${Ia\bar 3d}$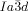 phase, contains only 3-way junctions. The key assumption is, as with the ${Ia\bar 3d}$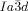 phase, that at the junctions, in order to interlock smoothly, the molecules twist with the same sense. As an example, a portion of the middle network forming a closed loop of 6 junctions is shown in the ribbon representation in Figure 4 f. There are three “perfect” (I) and three “imperfect” (II) junctions in that loop, detailed in Figures 4 g,h and i,j, respectively. In junctions I all ribbons merge perfectly smoothly, while in junctions II the blue and red ribbons merge smoothly but the green comes at an angle. We note that the latter defect is of little consequence because even if all green segments were disconnected, the remaining network would still remain connected and percolating the entire domain of the phase. The full extent of the middle network within the unit cell is shown in Figures 4 d,e,, with the Schwartz P minimum surface added in Figure 4 d. A calculation similar to that for the ${Ia\bar 3d}$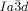 phase (details in SI) gives the average twist angle between adjacent molecules as 30–35°, that is, significantly higher than the value for the ${Ia\bar 3d}$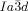 phase, and in line with the expectation.

A similar calculation can be performed on the other two networks; however because of their complexity we shall not discuss them here. The important point to make, however, is that the chirality of the middle network will undoubtedly affect the chirality of the inner and outer networks. Because of their crystallographic equivalence, the helical sense of the inner and outer (red and blue) network will be the same. Whether it will be the same or opposite of that of the middle network, we cannot tell with certainty. However, what is certain is that, in the general case, there will always be at least a residual chirality in the cubic ${{\it Im}\bar 3m}$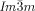 phase, as indeed observed.

The above model implies that each of the cubic structures is the solution best suited to accommodate a certain range of required molecular twist angles. It is not suggested that the ${{\it Im}\bar 3m}$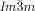 provides a general solution for any situation that requires high twist. In fact experiments on the homologous series of compounds **5** and **6** have shown[19] that as the terminal chains become very long, the ${{\it Im}\bar 3m}$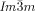 is again replaced by the ${Ia\bar 3d}$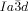. It would thus appear that such molecules require a twist exceeding the range covered by the ${{\it Im}\bar 3m}$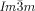 structure. In fact, one can speculate that for an intermolecular twist above 40° the exact match between the helical pitch and segment length ceases to dominate the choice of the phase, and the simpler ${Ia\bar 3d}$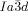 wins once again. Furthermore, due to lateral thermal expansion of the terminal chains, the twist is expected to increase on heating. This would explain the different temperature sequences observed for some homologues, where for the short chain compound an ${Ia\bar 3d}$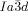-${{\it Im}\bar 3m}$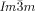 sequence is observed on heating, whereas for the long homologues this sequence is reversed (series of compounds **6**).[19]

A closer look at the Iso_LT_^[*]^-Cub transitions indicates that the germs of the achiral ${Ia\bar 3d}$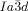 phase emerge always at the interface between the domains with opposite handedness. This appears to be favorable for the formation of this superstructure composed of two enantiomorphic networks (see Figure 2 e and Video 1b). In contrast, the germs of the ${{\it Im}\bar 3m}$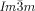 phase are not bound to the domain boundaries. The chiral sense of the nuclei once formed is retained and growth takes place preferentially within the same domain. But there is also a slower growth into domains with opposite chirality, leading to a change of the relative areas of the different domains (compare Figure 2 c,d, see also Video 1e). This means, that the growing Cub^[*]^/${{\it Im}\bar 3m}$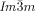 flips over the chirality of the liquid domains if required, although this causes a notable growth retardation.

In summary, we have discovered that, in spite of appearing in achiral compounds, the ${{\it Im}\bar 3m}$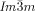 cubic LC phase is chiral in all compounds studied, while the ${Ia\bar 3d}$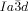 phase is invariably achiral.[30] This previously unrecognized features have led us to propose a model of the two phases based on networks with helical segments where the twist sense propagates in 3D across macroscopic domains through matching twist at network junctions. While the opposing chiralities of the two networks of the ${Ia\bar 3d}$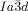 phase cancel, this cannot happen in the triple network ${{\it Im}\bar 3m}$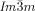 phase. This model also offers, for the first time, a feasible explanation for the existence of the complex ${{\it Im}\bar 3m}$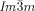 phase, and for the observed ${Ia\bar 3d}$
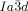
–${{\it Im}\bar 3m}$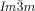 phase sequences as a function of chain length and temperature. The structural insight acquired herein promises to end one of the longest persisting mysteries in the liquid crystal field, that started with the discovery of the “smectic-D” phase over half a century ago.[31,32] But the importance of this finding goes far beyond the systems discussed herein and contributes to the general understanding of symmetry breaking in soft condensed matter.

## Notes

Supporting information for this article (syntheses, analytical data, DSC traces, additional crystallographic data and optical textures) as well as supporting videos (showing the growth of the Cub/ phase from the Iso[*] phase of compound 1b at T=1608C (Video_1b_160C) and of the Cub[*]/ from Iso[*] of compound 1e at T=1758C (Video_1e_175C) are available on the WWW under http://dx.doi.org/10.1002/anie.201406907.
