# Supplementary material for: Dynamic Mirror-Symmetry Breaking in Bicontinuous Cubic Phases
Source: Angew Chem Int Ed Engl. 2014 Sep 26;53(48):13115–20. doi: 10.1002/anie.201406907 (PMC4501316; doi:10.1002/anie.201406907)
Supplement: Supplementary file 1 [file anie0053-13115-sd1.pdf]

Angewandte Chemie
Eine Zeitschrift der Gesellschaft Deutscher Chemiker

Supporting Information

© Wiley-VCH 2014

69451 Weinheim, Germany

# Dynamic Mirror-Symmetry Breaking in Bicontinuous Cubic Phases**

*Christian Dressel, Feng Liu, Marko Prehm, Xiangbing Zeng,* Goran Ungar,* and Carsten Tschierske**

anie_201406907_sm_miscellaneous_information.pdf
anie_201406907_sm_Video_1b_160C.flv
anie_201406907_sm_Video_1e_175C.flv

**Video_1b_160C** shows the growth of the Ia3d phase in compound **1b** from the chiral isotropic phase $Iso_{LT}^{[*]}$ at 160°C. The sample is viewed in a microscope between slightly uncrossed polarizers. That is, the analyser is not at 90° to the polarizer, but at ±φ away from 90°, where φ is a small angle (several degrees). Soon after the start of the video, still in the $Iso_{LT}^{[*]}$ phase, the analyser is rotated from 90°-φ to 90°+φ. The contrast in the domains of opposite chiralities is seen to reverse, as the two domain types have equal but opposite optical activities. The Ia3d cubic phase is then seen to start nucleating; small polygonal (faceted) domains of intermediate brightness appear and grow. The nucleation happens invariably at the interface between the domains of opposite chiralities. Patches of the Ia3d phase grow into the two adjacent enantiomorphic domains at equal rate. Once the whole field of view had been converted to the Ia3d cubic, the analyser is turned back to the 90°-φ position. This time the contrast does not change, since the Ia3d phase is optically inactive.

**Video_1e_175C** shows the growth of the Im3m cubic phase from the chiral isotropic $Iso_{LT}^{[*]}$ liquid of compound **1e** at 175°C. Initially the analyser is slightly uncrossed at 90°+φ. The Im3m cubic phase nucleates outside the field of view. It enters the visible field at top right as a single dark domain, having the same shade as the dark $Iso_{LT}^{[*]}$ domains and thus having the same or very similar optical rotatory power as the chiral liquid. The $Iso_{LT}^{[*]}$ spreads across the entire field of view. Evidently the growth rate through the $Iso_{LT}^{[*]}$domains of the same chirality is significantly faster than that through domains of the opposite chirality. Also note that the $Iso_{LT}^{[*]}$/Im3m boundaries are curved rather than faceted. At the end the analyser is rotated from 90°+φ to 90°-φ; the field turns brighter, indicating that the Im3m cubic is indeed optically active.

# Content

**1. Materials** .................................................................................................... S3

**1.1 Synthesis and analytical data of intermediates 8–11** .................................. S3

**1.2 Synthesis and analytical data of the bithiophenes 1** ................................... S5

**1.3 Synthesis and analytical data of compound 2**................................................ S7

**1.4 Analytical data of compounds 3 and 4** ......................................................... S7

**1.5 Transition temperatures of compounds 2–7**................................................. S9

**2. DSC-Investigations**.......................................................................................... S10

**3. CD and UV/VIS spectroscopy** ......................................................................... S14

**4. X-Ray Diffraction**............................................................................................. S15

**4.1 X-ray scattering using laboratory source** ...................................................... S15

**4.2 Synchrotron X-ray diffraction and electron density reconstruction** ............... S15

**4.3 Powder diffraction patterns**.............................................................................. S16

**4.4 Tables with *d*-spacings and diffraction intensities** ...................................... S17

**4.5 2D Patterns** ....................................................................................................... S22

**5. Electron density maps**....................................................................................... S23

**6. Additional details of structural models** ............................................................ S24

**6.1 Framework models with minimum surface**...................................................... S24

**6.2 Calculation of number of molecules and the geometry of cubic networks**........ S24

**7. Optical microscopy**............................................................................................ S25

**8. Complete references with more than 10 authors in the main text**……………………...S29

**9. References** ......................................................................................................... S29

# 1. Materials

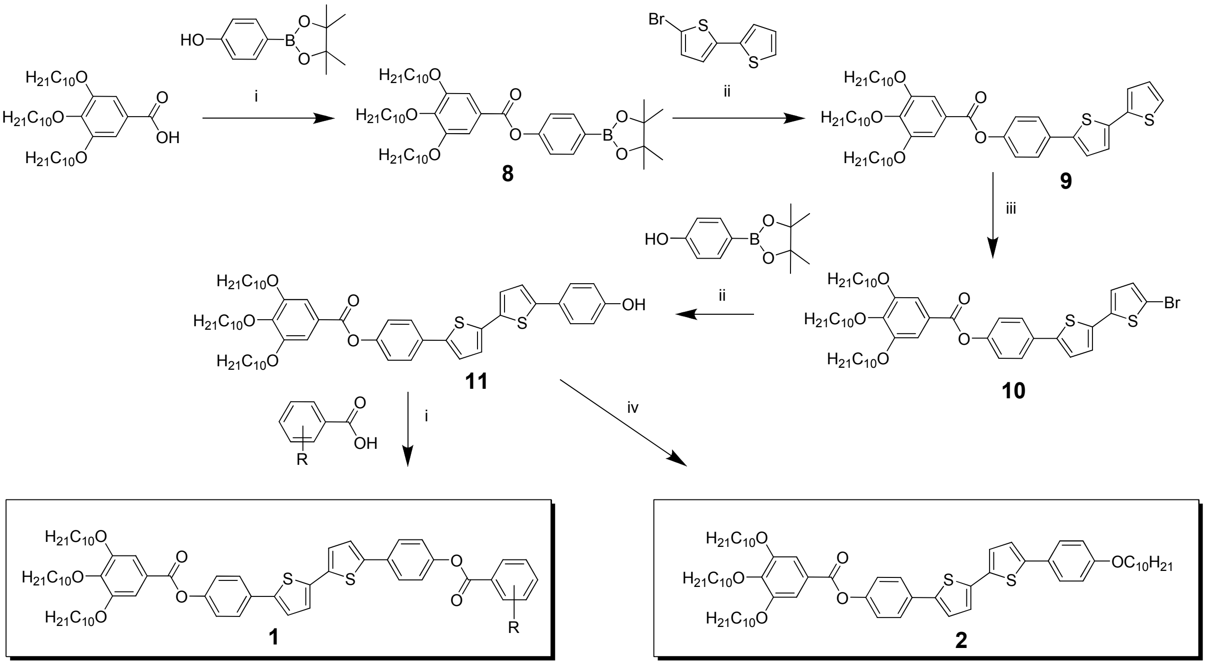


**Scheme S1**. Synthesis of compounds **1** and **2**. Reagents and conditions: (i) $SOCl_2$, abs. pyridine, RT; (ii) THF, sat. $NaHCO_3$ solution, $[Pd(PPh_3)_4]$, reflux; (iii) abs. THF, NBS, RT; (iv) abs. 2-butanone, *n*-$C_{10}H_{21}Br$, $K_2CO_3$; $N(n\text{-}C_4H_9)_4I$; reflux.

All reactions were carried out under argon. Dry solvents were purchased from commercial sources and used without further purification. NMR spectra were recorded on Varian Gemini 2000 or Unity 500 spectrometers at 27 °C with trimethylsilane as internal standard. Mass spectra were taken on Finnigan LCQ (electrospray, spray-voltage 6.7 kV, sheath gas nitrogen). Microanalyses were performed using a Leco CHNS-932 elemental analyser. Column chromatography was performed with Macherey-Nagel silica gel 60 (230–400 mesh). 4-Hydroxyphenylboronic acid pinacol ester was obtained from Sigma Aldrich and was used as obtained. 5-Bromo-2,2'-bithiophene was prepared according to reported standard procedures[S1] using THF as solvent[S2].

## 1.1 Synthesis and analytical data of intermediates 8–11

### 1.1.1 Synthesis of pinacol ester 8

3,4,5-Tri-*n*-decyloxybenzoic acid (3.2 g, 5.5 mmol) and $SOCl_2$ (20 ml) were refluxed for 30 minutes. $SOCl_2$ was removed under vacuum and dry pyridine (10 ml) and 4-hydroxyphenylboronic acid pinacol ester (1.2 g, 5.5 mmol) were added and the resulting mixture stirred at room temperature overnight. The solvent was evaporate and the residue was purified by column chromatography (eluent: $CH_2Cl_2$).

**8**: 4-(3,4,5-Tri-*n*-decyloxybenzoyloxy)phenylboronic acid pinacol ester; yield 2.7 g (3.4 mmol, 64%); colorless oil; $^1H$ NMR (400 MHz, $CDCl_3$): δ $^1H$-NMR (400 MHz, $CDCl_3$) δ 7.88 (d, $^3J$ = 8.4 Hz, 2H, Ar-H), 7.40 (s, 2H, Ar-H), 7.20 (d, $^3J$ = 8.4 Hz, 2H, Ar-H), 4.06 (t, $^3J$

= 6.6 Hz, 2H, $OCH_2CH_2$), 4.04 (t, $^3J$ = 6.5 Hz, 4H, $OCH_2CH_2$), 1.86–1.79 (m, 4H, $OCH_2CH_2$), 1.78–1.70 (m, 2H, $OCH_2CH_2$), 1.52–1.43 (m, 6H, $CH_2$), 1.35 (s, 12H, $CCH_3$), 1.40–1.21 (br, 36H, $CH_2$), 0.90–0.86 (m, 9H, $CH_3$).

### 1.1.2 Synthesis of 9 via Suzuki coupling reaction[S3]

A mixture of **8** (2.7 g, 3.4 mmol), 5-bromo-2,2'-bithiophene (0.8 g, 3.4 mmol) THF (120 ml) and saturated $NaHCO_3$ solution (60 ml) degassed with argon for 15 min. [$Pd(PPh_3)_4$] (0.2 g, 0.2 mmol) was added and the solution was refluxed for 4 h. After the reaction mixture was allowed to come to room temperature it was extracted twice with $CHCl_3$. The organic phase was dried over anhydrous $Na_2SO_4$, filtered and concentrated in vacuum. The crude product was purified by column chromatography (eluent: $CHCl_3$/*n*-hexane 1/1, v/v).

**9**: 5-[4-(3,4,5-Tri-*n*-decyloxybenzoyloxy)phenyl]-2,2'-bithiophene; 1.87 g (2.25 mmol, 66%); light yellow solid; m.p. 58 °C; $^1$H NMR (500 MHz, $CDCl_3$): δ 7.63 (d, $^3J$ = 8.6 Hz, 2H, Ar-H), 7.40 (s, 2H, Ar-H), 7.23–7.19 (m, 5H, Ar-H + Th-H), 7.14 (d, $^3J$ = 3.7 Hz, 1H, Th-H), 7.03 (dd, $^3J$ = 5.0 Hz, $^3J$ = 3.7 Hz, 1H, Th-H), 4.07 (t, $^3J$ = 6.5 Hz, 2H, $OCH_2CH_2$), 4.05 (t, $^3J$ = 6.5 Hz, 4H, $OCH_2CH_2$), 1.86–1.81 (m, 4H, $OCH_2CH_2$), 1.80–1.74 (m, 2H, $OCH_2CH_2$), 1.52–1.46 (m, 6H, $CH_2$), 1.41–1.22 (br, 36H, $CH_2$), 0.89 (t, $^3J$ = 7.0 Hz, 3H, $CH_3$), 0.88 (t, $^3J$ = 7.0 Hz, 6H, $CH_3$).

### 1.1.3 Synthesis of 10

Compound **9** (1.87 g, 2.25 mmol) dissolved in dry THF (200 ml). To the solution NBS (0.40 g, 2.25 mmol) was added in small portions at room temperature in the absence of light. The mixture was stirred for 3 h and then saturated $Na_2S_2O_3$ solution (20 ml) was added and the product was extracted two times with $CHCl_3$. The organic layer was washed with water and dried over anhydrous $Na_2SO_4$. After filtration the solvents were removed in vacuum and the crude product was purified by column chromatography (eluent: $CHCl_3$/*n*-hexane 1/1, v/v).

**10**: 5-Bromo-5'-[4-(3,4,5-tri-*n*-decyloxybenzoyloxy)phenyl]-2,2'-bithiophene; yield 1.66 g (1.82 mmol, 81%); light yellow solid; m.p. 83 °C; $^1$H NMR (400 MHz, $CDCl_3$): δ 7.63 (d, $^3J$ = 8.6 Hz, 2H, Ar-H), 7.41 (s, 2H, Ar-H), 7.22 (d, $^3J$ = 8.7 Hz, 2H, Ar-H), 7.20 (d, $^3J$ = 3.8 Hz, 1H, Th-H), 7.08 (d, $^3J$ = 3.8 Hz, 1H, Th-H), 6.99 (d, $^3J$ = 3.9 Hz, 1H, Th-H), 6.94 (d, $^3J$ = 3.8 Hz, 1H, Th-H), 4.06 (t, $^3J$ = 6.6 Hz, 2H, $OCH_2CH_2$), 4.05 (t, $^3J$ = 6.5 Hz, 4H, $OCH_2CH_2$), 1.88–1.79 (m, 4H, $OCH_2CH_2$), 1.78–1.73 (m, 2H, $OCH_2CH_2$), 1.51–1.45 (m, 6H, $CH_2$), 1.42–1.18 (br, 36H, $CH_2$), 0.93–0.84 (m, 9H, $CH_3$).

### 1.1.4 Synthesis of 11

According to procedure 1.1.2 starting from **10** 1.66 g (1.82 mmol) and 4-hydroxyphenylboronic acid pinacol ester 0.40 g (1.82 mmol); purification by column chromatography (eluent: $CHCl_3$) and crystallization from EtOH.

**11**: 5-[4-(3,4,5-Tri-*n*-decyloxybenzoyloxy)phenyl]-5'-(4-hydroxyphenyl)-2,2'-bithiophene; yield 0.92 g (1.00 mmol, 55%); yellow solid; m.p. 132 °C; $^1$H-NMR (400 MHz, $CDCl_3$) δ 7.64 (d, $^3J$ = 8.5 Hz, 2H, Ar-H), 7.49 (d, $^3J$ = 8.6 Hz, 2H, Ar-H), 7.41 (s, 2H, Ar-H), 7.24–7.19 (m, 3H, Ar-H + Th-H), 7.15 (d, $^3J$ = 3.9 Hz, 1H, Th-H), 7.14 (d, $^3J$ = 3.7 Hz, 1H, Th-H), 7.11 (d, $^3J$ = 3.7 Hz, 1H, Th-H), 6.85 (d, $^3J$ = 8.5 Hz, 2H, Ar-H), 4.83 (s, 1H, OH), 4.07 (t, $^3J$ = 6.7 Hz, 2H, $OCH_2CH_2$), 4.05 (t, $^3J$ = 6.5 Hz, 4H, $OCH_2CH_2$), 1.91–1.80 (m, 4H, $OCH_2CH_2$), 1.78–1.72 (m, 2H, $OCH_2CH_2$), 1.41–1.45 (m, 6H, $CH_2$), 1.41–1.19 (br, 36H, $CH_2$), 0.93–0.84 (m, 9H, $CH_3$).

### 1.2 Synthesis and analytical data of the bithiophenes 1

The compounds **1** were synthesized in analogy to procedure 1.1.1 starting from the corresponding benzoic acid.

**1a**: 5-[4-(3,4,5-Tri-*n*-decyloxybenzoyloxy)phenyl]-5'-(4-benzoyloxyphenyl)-2,2'-bithiophene; yield 37 mg (63%); purified by column chromatography (eluent: $CHCl_3$/*n*-hexane 2/1, v/v) and repeated crystallization from THF/EtOH; yellow solid; $^1$H NMR (500 MHz, $CDCl_3$): δ 8.23 (dd, $^3J$ = 7.0 Hz, $^4J$ = 1.5 Hz, 2H, Ar-H), 7.69–7.64 (m, 5H, Ar-H), 7.54 (dd, $^3J$ = 7.8 Hz, 2H, Ar-H), 7.42 (s, 2H, Ar-H), 7.29–7.22 (m, 6H, Ar-H + Th-H), 7.19 (d, $^3J$ = 3.7 Hz, 2H, Th-H), 4.07 (t, $^3J$ = 6.6 Hz, 2H, $OCH_2CH_2$), 4.06 (t, $^3J$ = 6.5 Hz, 4H, $OCH_2CH_2$), 1.89–1.80 (m, 4H, $OCH_2CH_2$), 1.79–1.74 (m, 2H, $OCH_2CH_2$), 1.51–1.47 (m, 6H, $CH_2$), 1.40–1.24 (br, 36H, $CH_2$), 0.91–0.86 (m, 9H, $CH_3$); $^{13}$C NMR (125 MHz, $CDCl_3$): δ 165.1 , 165.0 (C=O), 153.0, 150.5, 150.4, 143.2, 142.3, 142.3, 136.8, 131.9, 131.8, 130.2, 128.6, 126.7, 126.7, 124.6, 124.1, 124.0, 123.7, 122.3, 122.3, 108.7 (Ar-C + Th-C), 73.6, 69.3 ($OCH_2$), 31.9, 31.9, 30.4, 29.7, 29.7, 29.6, 29.6, 29.6, 29.4, 29.3, 29.3, 26.1, 26.1, 22.7, 22.7 ($CH_2$), 14.1 ($CH_3$); MS (ESI, $CH_2Cl_2$): $M$ = 1026.55 g mol$^{-1}$, $m/z$ (%) = 1026.45 ([M•]$^+$, 100); elemental analysis: calc. for $C_{64}H_{82}O_7S_2$: C 74.81%, H 8.04%, found C 74.91%, H 8.03%.

**1b**: 5-[4-(3,4,5-Tri-*n*-decyloxybenzoyloxy)phenyl]-5'-[4-(4-methoxybenzoyloxy)phenyl]-2,2'-bithiophene; purified by column chromatography (eluent: $CHCl_3$) and repeated crystallization from THF/EtOH; yield 76 mg (50%); yellow solid; $^1$H NMR (400 MHz, $CDCl_3$): δ 8.15 (d, $^3J$ = 9.2 Hz, 2H, Ar-H), 7.64 (d, $^3J$ = 8.8 Hz, 2H, Ar-H), 7.63 (d, $^3J$ = 8.8 Hz, 2H, Ar-H), 7.40 (s, 2H, Ar-H), 7.23–7.19 (m, 6H, Ar-H + Th-H), 7.16 (d, $^3J$ = 3.8 Hz, 2H, Th-H), 6.98 (d, $^3J$ = 8.8 Hz, 2H, Ar-H), 4.05 (t, $^3J$ = 6.6 Hz, 2H, $OCH_2CH_2$), 4.04 (t, $^3J$ = 6.5 Hz, 4H, $OCH_2CH_2$), 3.89 (s, 3H, $OCH_3$), 1.87–1.78 (m, 4H, $OCH_2CH_2$), 1.78–1.69 (m, 2H, $OCH_2CH_2$), 1.50–1.41 (m, 6H, $CH_2$), 1.38–1.21 (br, 36H, $CH_2$), 0.91–0.82 (m, 9H, $CH_3$); $^{13}$C NMR (100 MHz, $CDCl_3$): δ 164.9, 164.8 (C=O), 164.0, 153.0, 150.6, 150.5, 143.1, 142.4, 142.3, 136.8, 136.7, 132.3, 131.8, 131.7, 126.6, 124.6, 124.6, 124.0, 124.0, 123.7, 122.3, 122.3, 122.3, 121.7, 113.9, 108.7 (Ar-C + Th-C), 73.6, 69.3 ($OCH_2$), 55.5 ($OCH_3$), 31.9, 31.9, 30.3, 29.7, 29.7, 29.6, 29.6, 29.4, 29.3, 29.3, 26.1, 26.0, 22.7, 22.7 ($CH_2$), 14.1 ($CH_3$); MS (ESI, $CH_2Cl_2$): $M$ = 1056.56 g mol$^{-1}$, $m/z$ (%) = 1056.52 ([M•]$^+$, 100); elemental analysis: calc. for $C_{65}H_{84}O_8S_2$: C 73.83%, H 8.01%, found C 73.44%, H 7.93%.

**1c**: 5-[4-(3,4,5-Tri-*n*-decyloxybenzoyloxy)phenyl]-5'-[4-(3,4-dimethoxybenzoyloxy)-phenyl]-2,2'-bithiophene; purified by column chromatography (eluent: $CHCl_3$) and repeated

crystallization from THF/EtOH; yield 49 mg (26%); yellow solid; $^{1}$H NMR (400 MHz, $CDCl_3$): δ 7.86 (dd, $^{3}J$ = 8.4 Hz, $^{4}J$ = 2.0 Hz, 1H, Ar-H), 7.67 (d, $^{4}J$ = 2.0 Hz, 1H, Ar-H), 7.64 (d, $^{3}J$ = 8.8 Hz, 2H, Ar-H), 7.63 (d, $^{3}J$ = 8.8 Hz, 2H, Ar-H), 7.40 (s, 2H, Ar-H), 7.23–7.19 (m, 6H, Ar-H + Th-H), 7.17 (d, $^{3}J$ = 3.8 Hz, 2H, Th-H), 6.95 (d, $^{3}J$ = 8.6 Hz, 1H, Ar-H), 4.05 (t, $^{3}J$ = 6.6 Hz, 2H, O$CH_2$$CH_2$), 4.04 (t, $^{3}J$ = 6.5 Hz, 4H, O$CH_2$$CH_2$), 3.97 (s, 3H, $OCH_3$), 3.96 (s, 3H, $OCH_3$), 1.87–1.78 (m, 4H, $OCH_2$$CH_2$), 1.78–1.71 (m, 2H, $OCH_2$$CH_2$), 1.49–1.44 (m, 6H, $CH_2$), 1.38–1.21 (br, 36H, $CH_2$), 0.91–0.81 (m, 9H, $CH_3$); $^{13}$C NMR (100 MHz, $CDCl_3$): δ 164.9, 164.9 (C=O), 153.7, 153.0, 150.5, 148.9, 143.2, 142.3, 142.3, 136.8, 136.8, 131.8, 131.8, 126.7, 124.6, 124.5, 124.0, 123.7, 122.3, 121.8, 112.4, 110.4, 108.7 (Ar-C + Th-C), 73.6, 69.3 ($OCH_2$), 56.1 ($OCH_3$), 31.9, 31.9, 30.3, 29.7, 29.7, 29.6, 29.6, 29.4, 29.3, 29.3, 26.1, 26.0, 22.7, 22.7 ($CH_2$), 14.1 ($CH_3$); MS (ESI, $CH_2Cl_2$): $M$ = 1086.57 g mol$^{-1}$, $m/z$ (%) = 1086.49 ([M•]$^+$, 100); elemental analysis: calc. for $C_{66}H_{86}O_9S_2$: C 72.89%, H 7.97%, found C 72.64%, H 7.99%.

**1e**: 5-[4-(3,4,5-Tri-*n*-decyloxybenzoyloxy)phenyl]-5'-[4-(4-decyloxybenzoyloxy)phenyl]-2,2'-bithiophene; purified by column chromatography (eluent: $CHCl_3$/*n*-hexane 1/1, v/v) and repeated crystallization from THF/EtOH; yield 107 mg (46%); yellow solid; $^{1}$H NMR (400 MHz, $CDCl_3$): δ 8.15 (d, $^{3}J$ = 8.9 Hz, 2H, Ar-H), 7.66 (d, $^{3}J$ = 8.4, 2H, Ar-H), 7.65 (d, $^{3}J$ = 8.8 Hz, 2H, Ar-H), 7.42 (s, 2H, Ar-H), 7.25–7.21 (m, 6H, Ar-H + Th-H), 7.18 (d, $^{3}J$ = 3.8 Hz, 2H, Th-H), 6.98 (d, $^{3}J$ = 8.9 Hz, 2H, Ar-H), 4.08–4.04 (m, 8H, O$CH_2$$CH_2$), 1.87–1.80 (m, 6H, $OCH_2$$CH_2$), 1.79–1.73 (m, 2H, $OCH_2$$CH_2$), 1.51–1.44 (m, 8H, $CH_2$), 1.40–1.22 (br, 48H, $CH_2$), 0.95–0.80 (m, 12H, $CH_3$); $^{13}$C NMR (100 MHz, $CDCl_3$): δ 164.9, 164.9 (C=O), 163.6, 153.0, 150.6, 150.5, 143.1, 142.4, 142.2, 136.8, 136.7, 132.3, 131.8, 131.7, 126.7, 124.6, 124.6, 124.0, 124.0, 123.7, 122.3, 122.3, 121.4, 114.3, 108.6 (Ar-C + Th-C), 73.6, 69.3, 68.4 ($OCH_2$), 31.9, 31.9, 31.9, 30.3, 29.7, 29.7, 29.6, 29.6, 29.6, 29.5, 29.5, 29.4, 29.3, 29.3, 29.1, 26.1, 26.0, 26.0, 22.7, 22.7 ($CH_2$), 14.1 ($CH_3$); MS (ESI, $CH_2Cl_2$): $M$ = 1182.70 g mol$^{-1}$, $m/z$ (%) = 1182.62 ([M•]$^+$, 100); elemental analysis: calc. for $C_{74}H_{102}O_8S_2$: C 75.08%, H 8.69%, found C 75.16%, H 8.94%.

**1f**: 5-[4-(3,4,5-Tri-*n*-decyloxybenzoyloxy)phenyl]-5'-[4-(3-ethoxybenzoyloxy)phenyl]-2,2'-bithiophene; purified by column chromatography (eluent: $CHCl_3$) and repeated crystallization from THF/EtOH; yield 92 mg (86%); yellow solid; $^{1}$H NMR (400 MHz, $CDCl_3$): δ 7.81 (ddd, $^{3}J$ = 7.6 Hz, $^{4}J$ = 1.6 Hz, $^{4}J$ = 1.0 Hz, 1H, Ar-H), 7.71 (dd, $^{4}J$ = 2.5 Hz, $^{4}J$ = 1.6 Hz, 1H, Ar-H), 7.66 (d, $^{3}J$ = 8.6 Hz, 2H, Ar-H), 7.65 (d, $^{3}J$ = 8.6 Hz, 2H, Ar-H), 7.42 (dd, $^{3}J$ = 7.6 Hz, 1H, Ar-H), 7.41 (s, 2H, Ar-H), 7.27–7.22 (m, 6H, Ar-H + Th-H), 7.19 (d, $^{3}J$ = 3.8 Hz, 2H, Th-H), 7.18 (ddd, $^{3}J$ = 7.6 Hz, $^{4}J$ = 2.5 Hz, $^{4}J$ = 1.0 Hz, 1H, Ar-H), 4.13 (q, $^{3}J$ = 7.0 Hz, 2H, O$CH_2$$CH_3$), 4.07 (t, $^{3}J$ = 6.4 Hz, 2H, O$CH_2$$CH_2$), 4.06 (t, $^{3}J$ = 6.4 Hz, 4H, O$CH_2$$CH_2$), 1.88–1.81 (m, 4H, $OCH_2$$CH_2$), 1.79–1.73 (m, 2H, $OCH_2$$CH_2$), 1.51–1.48 (m, 6H, $CH_2$), 1.46 (t, $^{3}J$ = 7.0 Hz, 3H, $OCH_2$$CH_3$), 1.40–1.24 (br, 36H, $CH_2$), 0.92–0.85 (m, 9H, $CH_3$); $^{13}$C NMR (125 MHz, $CDCl_3$): δ 165.0, 165.0 (C=O), 153.0, 150.5, 150.4, 143.1, 142.3, 142.3, 136.8, 136.8, 131.9, 131.8, 130.6, 129.6, 126.7, 126.7, 124.6, 124.1, 124.0, 123.7, 122.5, 122.3, 122.3, 120.7, 115.2, 108.6 (Ar-C + Th-C), 73.6, 69.3 ($OCH_2$), 63.8 (O$CH_2$$CH_3$), 31.9, 31.9, 30.3, 29.7, 29.7, 29.6, 29.6, 29.6, 29.4, 29.3, 29.3, 26.1, 26.1, 22.7, 22.7 ($CH_2$), 14.7 ($OCH_2$$CH_3$), 14.1 ($CH_3$); MS (ESI, $CH_2Cl_2$): $M$ = 1070.58 g mol$^{-1}$, $m/z$ (%) = 1070.59 ([M•]$^+$, 100); elemental analysis: calc. for $C_{66}H_{86}O_8S_2$: C 73.98%, H 8.09%, found C 74.41%, H 8.34%.

**1g**: 5-[4-(3,4,5-Tri-*n*-decyloxybenzoyloxy)phenyl]-5'-[4-(3,4,5-trimethoxybenzoyloxy)-phenyl]-2,2'-bithiophene; purified by column chromatography (eluent: $CHCl_3$) and repeated crystallization from THF/EtOH; yield 59 mg (84%); yellow solid; $^1$H NMR (400 MHz, $CDCl_3$): δ 7.67 (d, $^3J$ = 8.4, 2H, Ar-H), 7.66 (d, $^3J$ = 8.4, 2H, Ar-H), 7.47 (s, 2H, Ar-H), 7.42 (s, 2H, Ar-H), 7.25–7.21 (m, 6H + Th-H), 7.19 (d, $^3J$ = 3.8 Hz, 2H, Th-H), 4.07 (t, $^3J$ = 6.6 Hz, 2H, OC$H_2$CH$_2$), 4.06 (t, $^3J$ = 6.5 Hz, 4H, OC$H_2$CH$_2$), 3.96 (s, 9H, $OCH_3$), 1.88–1.80 (m, 4H, OCH$_2$C$H_2$), 1.79–1.73 (m, 2H, OCH$_2$C$H_2$), 1.51–1.46 (m, 6H, $CH_2$), 1.41–1.22 (br, 36H, $CH_2$), 0.92–0.85 (m, 9H, $CH_3$); $^{13}$C NMR (100 MHz, $CDCl_3$): δ 165.0, 164.8 (C=O), 153.1, 153.0, 150.5, 150.4, 143.1, 142.3, 136.8, 136.8, 131.8, 126.7, 126.7, 124.6, 124.2, 124.1, 124.0, 123.7, 122.3, 122.3, 108.6, 107.5 (Ar-C + Th-C), 73.6, 69.3 ($OCH_2$), 61.0, 56.4 ($OCH_3$), 31.9, 31.9, 30.3, 29.7, 29.7, 29.6, 29.6, 29.4, 29.3, 29.3, 26.1, 26.0, 22.7, 22.7 ($CH_2$), 14.1 ($CH_3$); MS (ESI, $CH_2Cl_2$): *M* = 1116.58 g mol$^{-1}$, *m/z* (%) = 1116.51 ([M•]$^+$, 100); elemental analysis: calc. for $C_{67}H_{88}O_{10}S_2$: C 72.01%, H 7.94%, found C 71.84%, H 7.85%.

## 1.3 Synthesis and analytical data of compound 2

Phenol **11** (142 mg, 0.15 mmol), 1-decylbromide (37 mg, 0.17 mmol), $K_2CO_3$ (104 mg, 0.75 mmol) and catalytic amounts of tetra-*n*-butylammoniumiodide were dissolved in dry 2-butanone (20 ml) and the mixture refluxed for 6 h. The solvent was removed under vacuum and the crude product was purified by column chromatography (eluent: $CHCl_3$/*n*-hexane 3/2, v/v) and repeated crystallization from THF/EtOH.

**2**: 5-[4-(3,4,5-Tri-*n*-decyloxybenzoyloxy)phenyl]-5'-(4-decyloxyphenyl)-2,2'-bithiophene; yield 41 mg (25%); yellow solid; $^1$H NMR (400 MHz, $CDCl_3$): δ 7.63 (d, $^3J$ = 8.7 Hz, 2H, Ar-H), 7.50 (d, $^3J$ = 8.4 Hz, 2H, Ar-H), 7.40 (s, 2H, Ar-H), 7.22–7.19 (m, 3H, Ar-H), 7.13 (d, $^3J$ = 3.8 Hz, 2H, Th-H), 7.10 (d, $^3J$ = 3.8 Hz, 1H, Th-H), 6.90 (d, $^3J$ = 8.8 Hz, 2H, Ar-H), 4.05 (t, $^3J$ = 6.5 Hz, 2H, OC$H_2$CH$_2$), 4.04 (t, $^3J$ = 6.5 Hz, 4H, OC$H_2$CH$_2$), 3.97 (t, $^3J$ = 6.6 Hz, 2H, OC$H_2$CH$_2$), 1.86–1.71 (m, 8H, OCH$_2$C$H_2$), 1.50–1.40 (m, 8H, $CH_2$), 1.36–1.21 (br, 48H, $CH_2$), 0.88–0.86 (m, 12H, $CH_3$); $^{13}$C NMR (125 MHz, $CDCl_3$): δ 165.0 (C=O), 159.0, 153.0, 150.5, 143.6, 143.2, 141.9, 137.2, 135.5, 131.9, 126.9, 126.7, 124.6, 124.2, 124.0, 123.8, 122.7, 122.3, 115.0, 108.6 (Ar-C + Th-C), 73.6, 69.3, 68.2 ($OCH_2$), 32.0, 31.9, 31.9, 30.4, 29.8, 29.7, 29.7, 29.6, 29.6, 29.6, 29.4, 29.4, 29.3, 29.3, 26.1, 26.1, 26.1, 22.7, 22.7 ($CH_2$), 14.1 ($CH_3$); MS (ESI, $CH_2Cl_2$): *M* = 1062.68 g mol$^{-1}$, *m/z* (%) = 1062.64 ([M•]$^+$, 100); elemental analysis: calc. for $C_{67}H_{98}O_6S_2$: C 75.66%, H 9.29%, found C 75.59%, H 9.52%.

## 1.4 Analytical data of compounds 3 and 4

**3**: 4-(3,4,5-Tri-*n*-decyloxybenzoyloxy)-4'-[4-(4-*n*-decyloxybenzoyloxy)benzoyloxy]biphenyl; synthesized by esterification of 4'-(3,4,5-tri-*n*-decyloxybenzoyloxy)biphenyl-4-ol[S4] with 4-(4-*n*-decyloxybenzoyloxy)benzoic acid as described in procedure 1.1.1; purified by column chromatography (eluent: $CH_2Cl_2$) and repeated crystallization from THF/EtOH; yield 95 mg (60%); white solid; $^1$H NMR (400 MHz, $CDCl_3$): δ 8.32 (d, $^3J$ = 8.8 Hz, 2H, Ar-H), 8.18 (d, $^3J$ = 8.9 Hz, 2H, Ar-H), 7.67 (d, $^3J$ = 8.7 Hz, 2H, Ar-H), 7.66 (d, $^3J$ = 8.6 Hz, 2H, Ar-H), 7.45 (s, 2H, Ar-H), 7.40 (d, $^3J$ = 8.8 Hz, 2H, Ar-H), 7.33 (d, $^3J$ = 8.6 Hz, 2H, Ar-H), 7.30 (d, $^3J$ =

8.6 Hz, 2H, Ar-H), 7.01 (d, $^3J$ = 8.9 Hz, 2H, Ar-H), 4.08 (t, $^3J$ = 6.6 Hz, 2H, OC$H_2$CH$_2$), 4.07 (t, $^3J$ = 6.5 Hz, 6H, OC$H_2$CH$_2$), 1.89–1.82 (m, 6H, OCH$_2$C$H_2$), 1.81–1.74 (m, 2H, OCH$_2$C$H_2$), 1.54–1.45 (m, 8H, CH$_2$), 1.45–1.21 (br, 48H, CH$_2$), 0.93–0.87 (m, 12H, CH$_3$); $^{13}$C NMR (100 MHz, CDCl$_3$): δ 165.0, 164.5, 164.3 (C=O), 163.8, 155.4, 153.0, 150.6, 150.4, 143.1, 138.3, 138.1, 132.4, 131.8, 128.2, 128.2, 126.8, 123.8, 122.1, 122.0, 121.0, 114.4, 108.6 (Ar-C), 73.6, 69.3, 68.4 (OCH$_2$), 31.9, 31.9, 31.9, 30.3, 29.7, 29.7, 29.6, 29.6, 29.6, 29.5, 29.5, 29.4, 29.3, 29.3, 29.3, 29.1, 26.1, 26.1, 26.0, 22.7, 22.7 (CH$_2$), 14.1 (CH$_3$); MS (ESI, CH$_2$Cl$_2$/MeOH): $M$ = 1138.75 g mol$^{-1}$, $m/z$ (%) = 1145.76 ([M+Li]$^+$, 100); elemental analysis: calc. for $C_{73}H_{102}O_{10}$: C 76.94%, H 9.02%, found C 77.17%, H 9.08%.

**4**: 4-(3,4,5-Tri-*n*-decyloxybenzoyloxy)-4'-[4-(4-*n*-decyloxyphenyl)benzoyloxy]biphenyl; synthesized by esterification of 4'-(3,4,5-tri-*n*-decyloxybenzoyloxy)biphenyl-4-ol[S4] with 4-(4-*n*-decyloxyphenyl)benzoic acid as described in procedure 1.1.1; purified by column chromatography (eluent: CH$_2$Cl$_2$) and repeated crystallization from THF/EtOH; yield 114 mg (78%); white solid; $^1$H NMR (500 MHz, CDCl$_3$): δ 8.28 (d, $^3J$ = 8.5 Hz, 2H, Ar-H), 7.72 (d, $^3J$ = 8.5 Hz, 2H, Ar-H), 7.67 (d, $^3J$ = 8.6 Hz, 2H, Ar-H), 7.66 (d, $^3J$ = 8.6 Hz, 2H, Ar-H), 7.62 (d, $^3J$ = 8.8 Hz, 2H, Ar-H), 7.45 (s, 2H, Ar-H), 7.34 (d, $^3J$ = 8.6 Hz, 2H, Ar-H), 7.30 (d, $^3J$ = 8.6 Hz, 2H, Ar-H), 7.03 (d, $^3J$ = 8.8 Hz, 2H, Ar-H), 4.09 (t, $^3J$ = 6.5 Hz, 2H, OC$H_2$CH$_2$), 4.08 (t, $^3J$ = 6.5 Hz, 4H, OC$H_2$CH$_2$), 4.04 (t, $^3J$ = 6.6 Hz, 2H, OC$H_2$CH$_2$), 1.90–1.82 (m, 6H, OCH$_2$C$H_2$), 1.81–1.75 (m, 2H, OCH$_2$C$H_2$), 1.54–1.45 (m, 8H, CH$_2$), 1.44–1.23 (br, 48H, CH$_2$), 0.93–0.88 (m, 12H, CH$_3$); $^{13}$C NMR (100 MHz, CDCl$_3$): δ 165.2, 165.0 (C=O), 159.6, 153.0, 150.6, 150.5, 146.1, 143.1, 138.2, 132.0, 130.7, 128.4, 128.2, 127.4, 126.6, 123.8, 122.1, 122.1, 115.0, 108.6 (Ar-C), 73.6, 69.3, 68.2 (CH$_2$), 31.9, 31.9, 31.9, 30.3, 29.7, 29.7, 29.6, 29.6, 29.4, 29.3, 29.3, 29.2, 26.1, 26.1, 26.0, 22.7, 22.7 (CH$_2$), 14.1 (CH$_3$); MS (ESI, CH$_2$Cl$_2$/MeOH): $M$ = 1094.76 g mol$^{-1}$, $m/z$ (%) = 1101.84 ([M+Li]$^+$, 100); elemental analysis: calc. for $C_{72}H_{102}O_8$: C 78.93%, H 9.38%, found C 78.98%, H 9.33%.

## 1.5 Transition temperatures of compounds 2–7

**Table S1.** Chemical structure and phase transitions (heating top lines, cooling bottom lines) and cubic lattice parameters of the compounds **2**–**7**.[a]

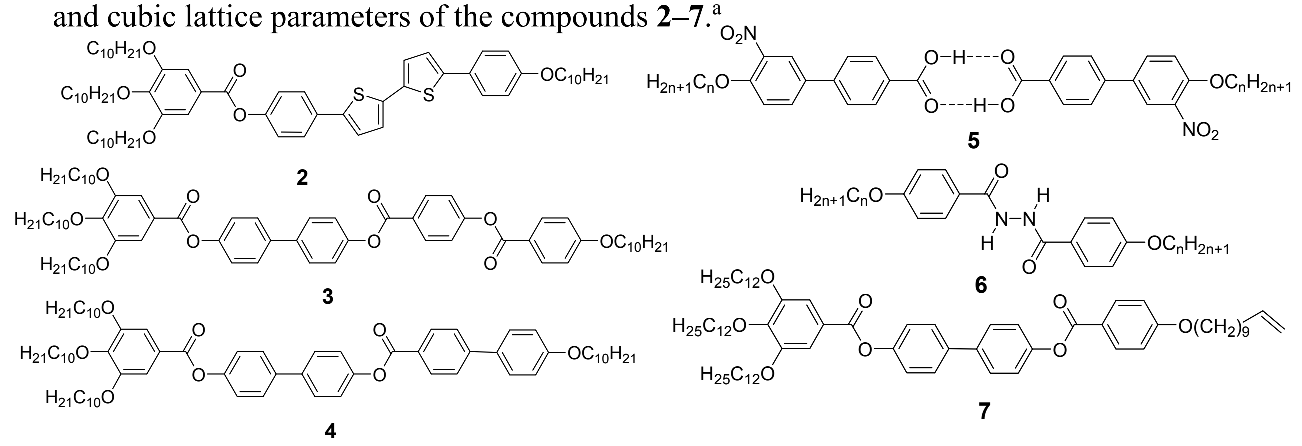


| Comp. | Phase transitions on heating/cooling ($T$/°C) | $a_{cub}$/nm | Ref. |
|---|---|---|---|
| **2** | Cr 67 [25.7] Cub[*]/ $Im\bar{3}m$ 123 [2.7] $Iso_{HT}$<br>$Iso_{HT}$ 119 [0.3] $Iso_{LT}$[*] 116 [1.3] Cub[*]/ $Im\bar{3}m$ | 16.49 | - |
| **3** | Cr 77 [35.5] Cub[*]/ $Im\bar{3}m$ 153 [1.7] $Iso_{HT}$<br>$Iso_{HT}$ 152 [0.7] $Iso_{LT}$[*] 143 [0.8] Cub[*]/ $Im\bar{3}m$ | 15.73 | - |
| **4** | Cr 78 [55.7] Cub[*]/ $Im\bar{3}m$ 162 [2.2] $Iso_{HT}$<br>$Iso_{HT}$ 158 [0.2] $Iso_{LT}$[*] 153 [1.4] Cub[*]/ $Im\bar{3}m$ | 16.30 | - |
| **5a**<br>($n$ = 16)[b] | Cr 127 [37.7] SmC 177 [0.5] Cub/ $Ia\bar{3}d$ 198 [1.0] SmA 200 [0.8] $Iso_{LT}$ 205 [0.6] $Iso_{HT}$<br>$Iso_{HT}$ 205 [1.4] $Iso_{LT}$ 198 [0.9] SmA 191 [1.1] Cub/ $Ia\bar{3}d$ 161 [0.6] SmC 98 [37.5] Cr | 10.76 | S5<br>S6 |
| **5b**<br>($n$ = 18)[b] | Cr 128 [43.6] SmC 164 [0.9] Cub/ $Ia\bar{3}d$ ~180 Cub[*]/ $Im\bar{3}m$ 199 [2.8] $Iso_{LT}$ 205 [0.9] $Iso_{HT}$<br>$Iso_{HT}$ 205 [2.1] $Iso_{LT}$ 190 [1.5] Cub/ $Ia\bar{3}d$ 99 [42.5] Cr | 11.39<br>( $Ia\bar{3}d$ )<br>16.88<br>( $Im\bar{3}m$ ) | S5<br>S6 |
| **5c**<br>($n$ = 20)[b] | Cr 113 [42.9] SmC 150 [1.2] Cub[*]/ $Im\bar{3}m$ 198 [2.5] $Iso_{LT}$ ~203 [–] $Iso_{HT}$<br>$Iso_{HT}$ ~203 [–] $Iso_{LT}$ 191 [2.0] Cub[*]/ $Im\bar{3}m$ 115 [1.6] SmC 56 [7.5] Cr | 17.59 | S5 |
| **6a**<br>($n$ = 7)[b] | Cr 143 [19.9] Cub/ $Ia\bar{3}d$ 164 [10.8] $Iso_{HT}$<br>$Iso_{HT}$ 162 [10.2] Cub/ $Ia\bar{3}d$ 130 [18.8] Cr | 6.09 | S7 |
| **6b**<br>($n$ = 10)[b] | Cr 143 [25.0] Cub/ $Ia\bar{3}d$ 154 [0.5] SmC 166 [12.1] $Iso_{HT}$<br>$Iso_{HT}$ 164 [11.4] SmC 143 [0.3] Cub/ $Ia\bar{3}d$ 133 [23.7] Cr | 7.04 | S7 |
| **6c**<br>($n$ = 14)[b] | Cr 128 [56.5] Cub[*]/ $Im\bar{3}m$ 159 [5.9] $Iso_{HT}$<br>$Iso_{HT}$ 154 [5.5] Cub[*]/ $Im\bar{3}m$ 120 [59.7] Cr | 12.22 | S7 |
| **7** | Cr 54 [47.1] Cub[*]/ $Im\bar{3}m$ 73 [1.7] $Iso_{HT}$<br>$Iso_{HT}$ ~67 [–] $Iso_{LT}$[*] 66 [0.9] Cub[*]/ $Im\bar{3}m$ 35 [51.6] Cr | 16.8 | S8 |

[a] Abbreviations: SmC = tilted smectic LC phase, SmA = nontilted smectic LC phase, $Iso_{LT}$ = achiral isotropic phase with cybotactic cluster structure; for the other abbreviations, see Table 1; [b] data from optical and DSC analysis of the resynthesized materials, the phase transitions are in a range of ±1 K identical with those reported in the given references, also the phase assignment by optical microscopy is in full agreement with that obtained with XRD investigations in the references.

## 2. DSC-Investigations

Differential scanning calorimetry was done using a Perkin Elmer DSC-7 instrument. Typical heating and cooling rates were 10 K min$^{-1}$.

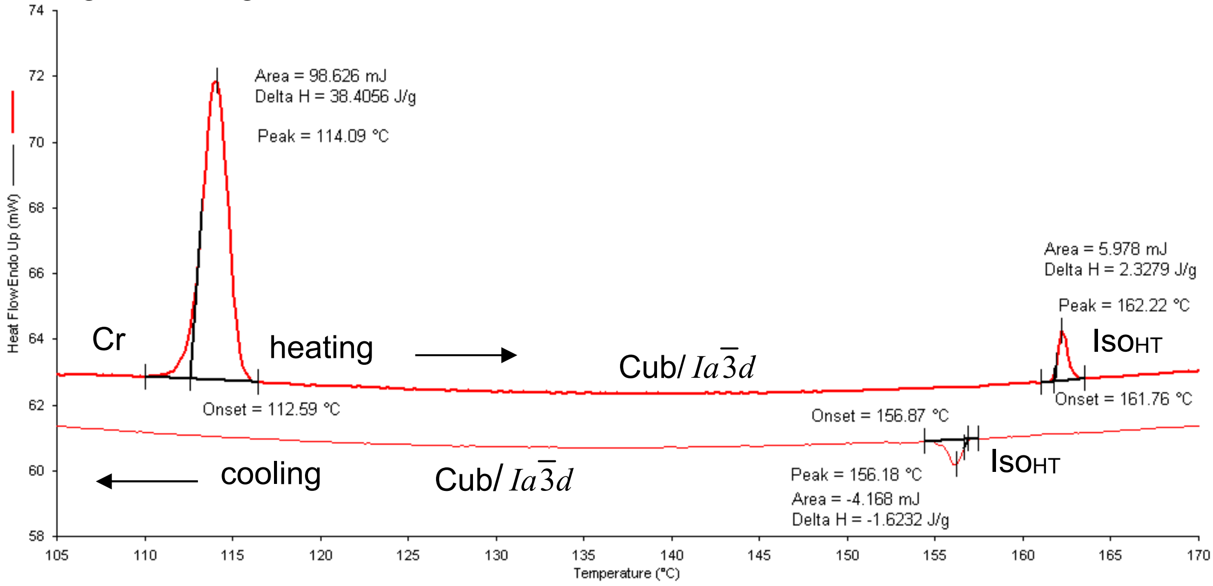


**Figure S1.** Section of the DSC heating and cooling scans (10 K min$^{-1}$) of compound **1a** in the temperature range between 105 and 170 °C.

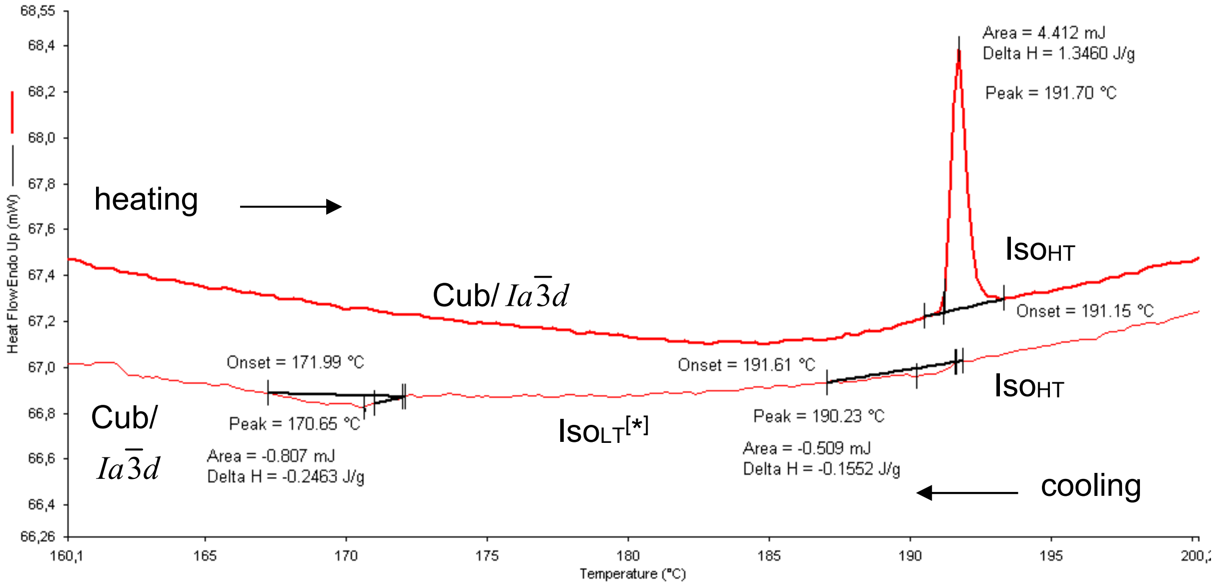


**Figure S2.** Section of the DSC heating and cooling scans (10 K min$^{-1}$) of compound **1b** in the temperature range between 160 and 200 °C.

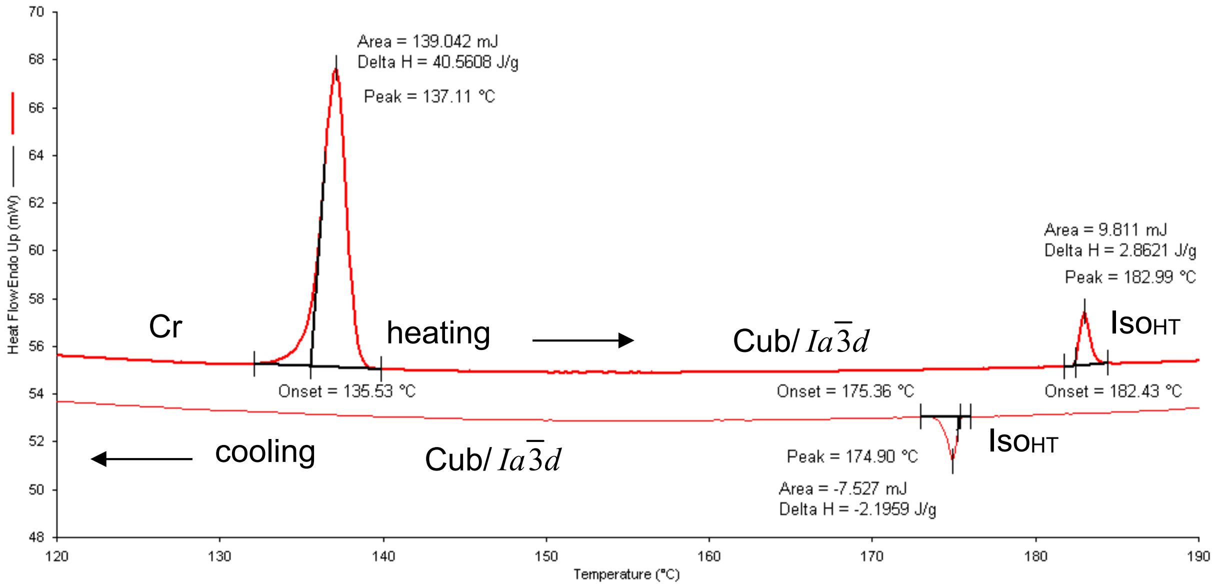


**Figure S3.** Section of the DSC heating and cooling scans (10 K min$^{-1}$) of compound **1c** in the temperature range between 120 and 190 °C.

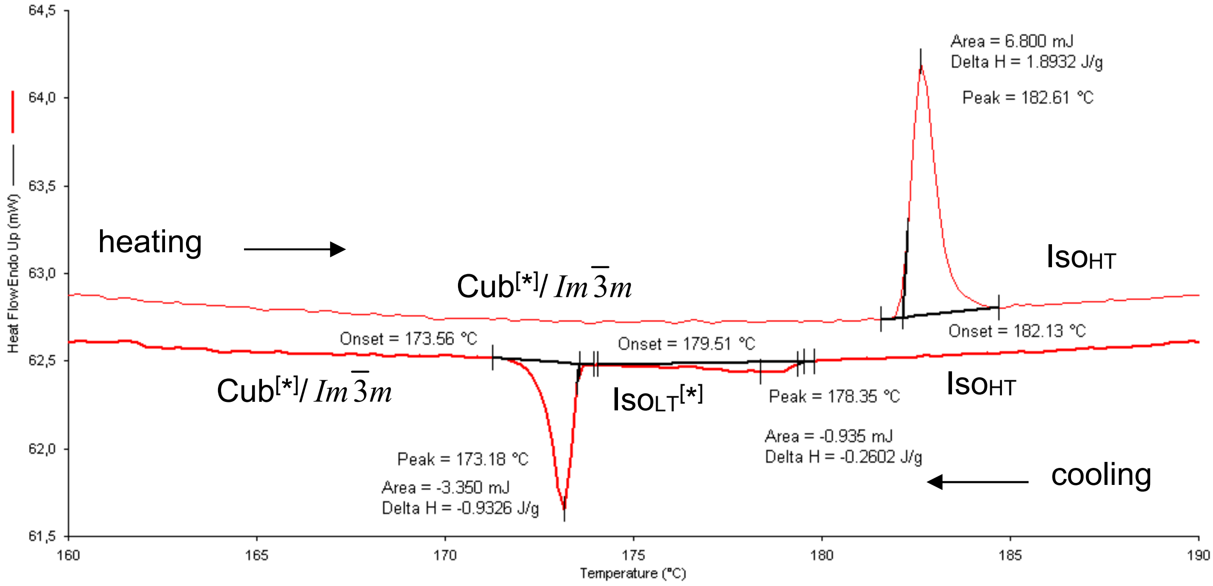


**Figure S4.** Section of the DSC heating and cooling scans (10 K min$^{-1}$) of compound **1e** in the temperature range between 160 and 190 °C.

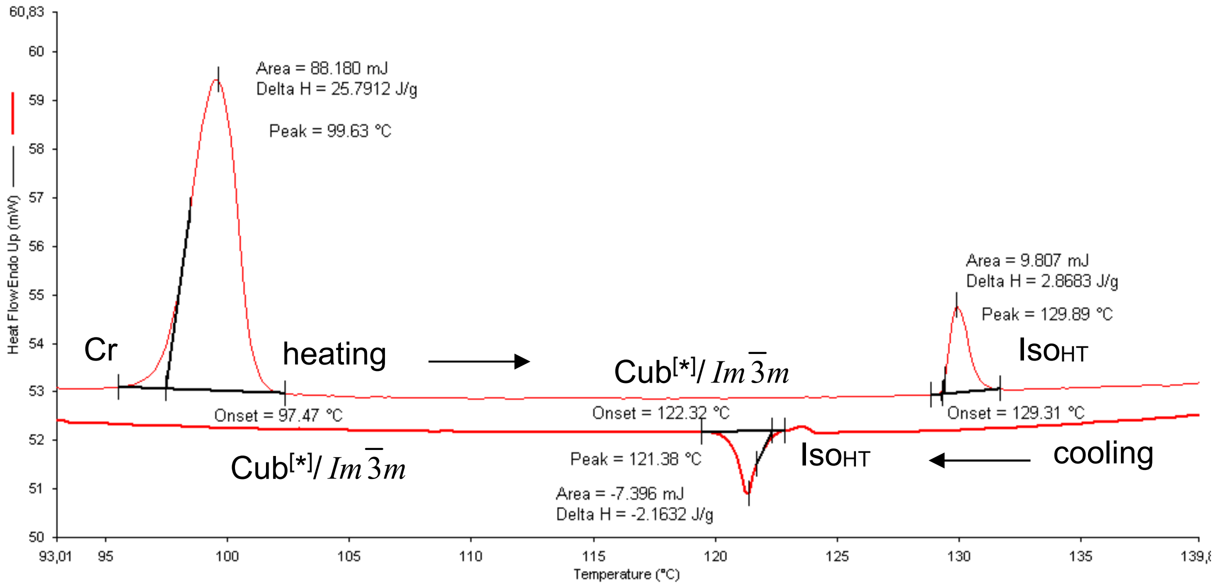


**Figure S5.** Section of the DSC heating and cooling scans (10 K min$^{-1}$) of compound **1f** in the temperature range between 93 and 139 °C.

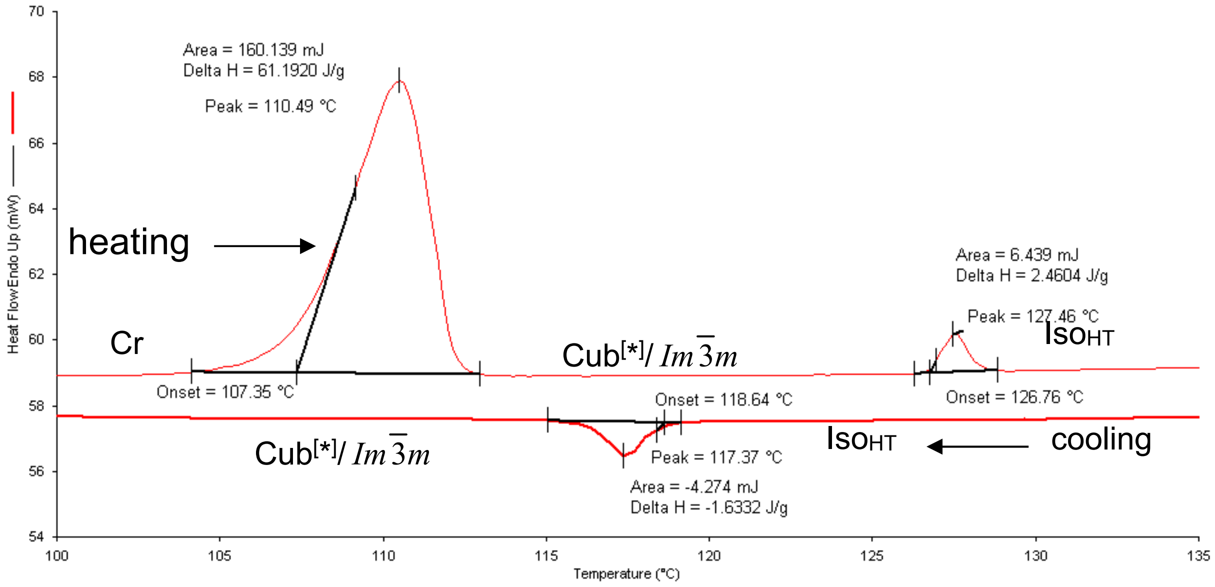


**Figure S6.** Section of the DSC heating and cooling scans (10 K min$^{-1}$) of compound **1g** in the temperature range between 100 and 135 °C.

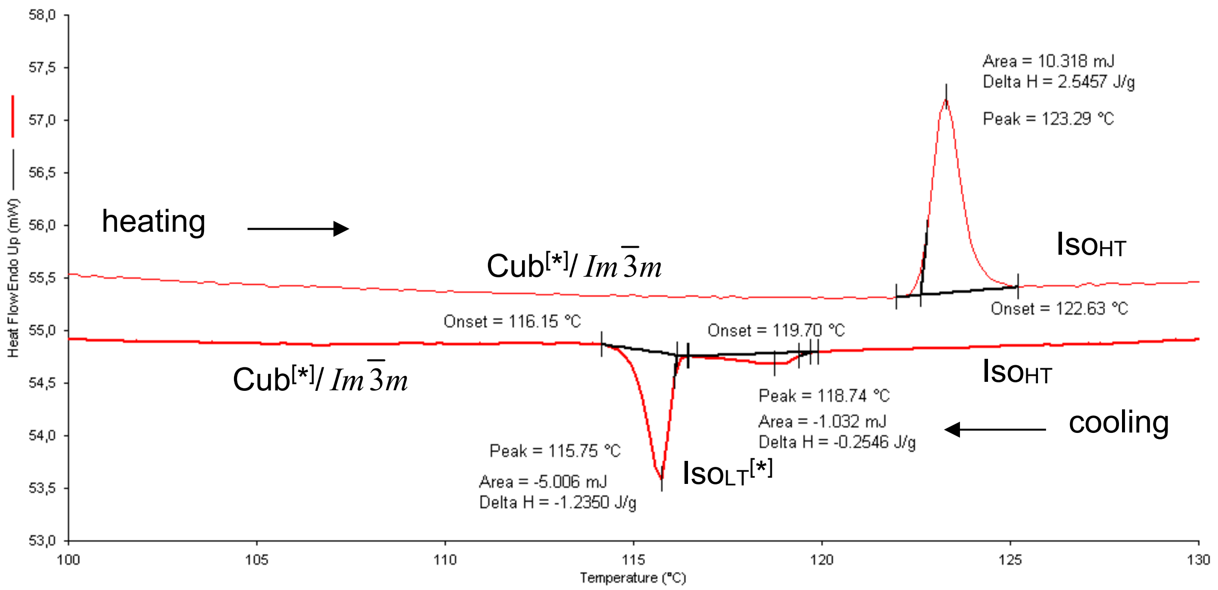


**Figure S7.** Section of the DSC heating and cooling scans (10 K min$^{-1}$) of compound **2** in the temperature range between 100 and 130 °C.

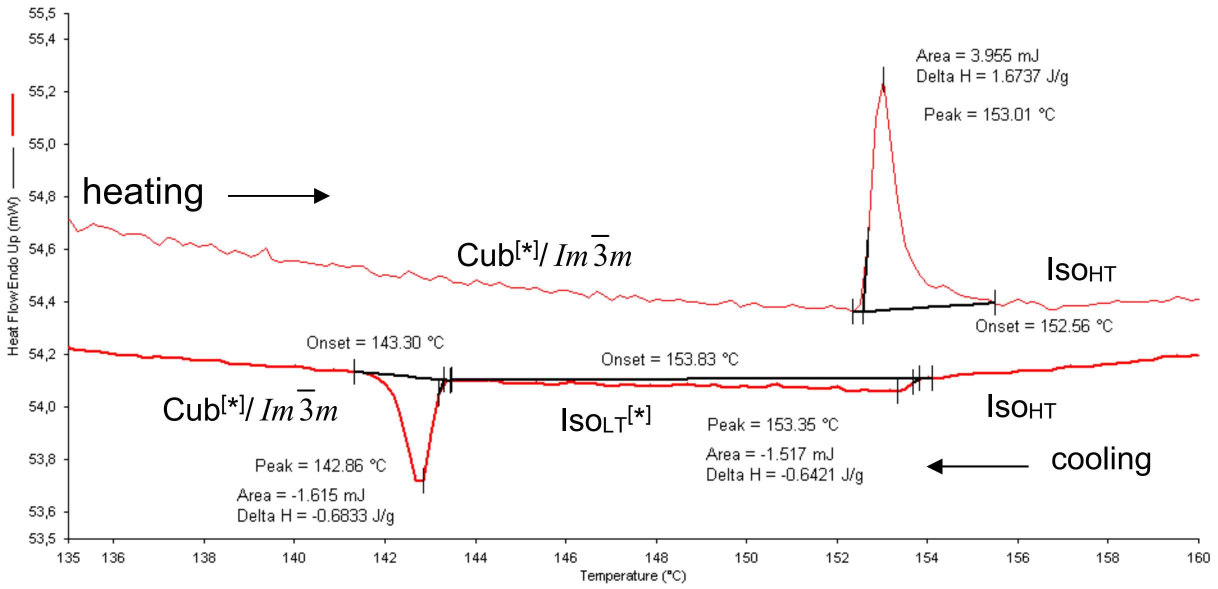


**Figure S8.** Section of the DSC heating and cooling scans (10 K min$^{-1}$) of compound **3** in the temperature range between 135 and 160 °C.

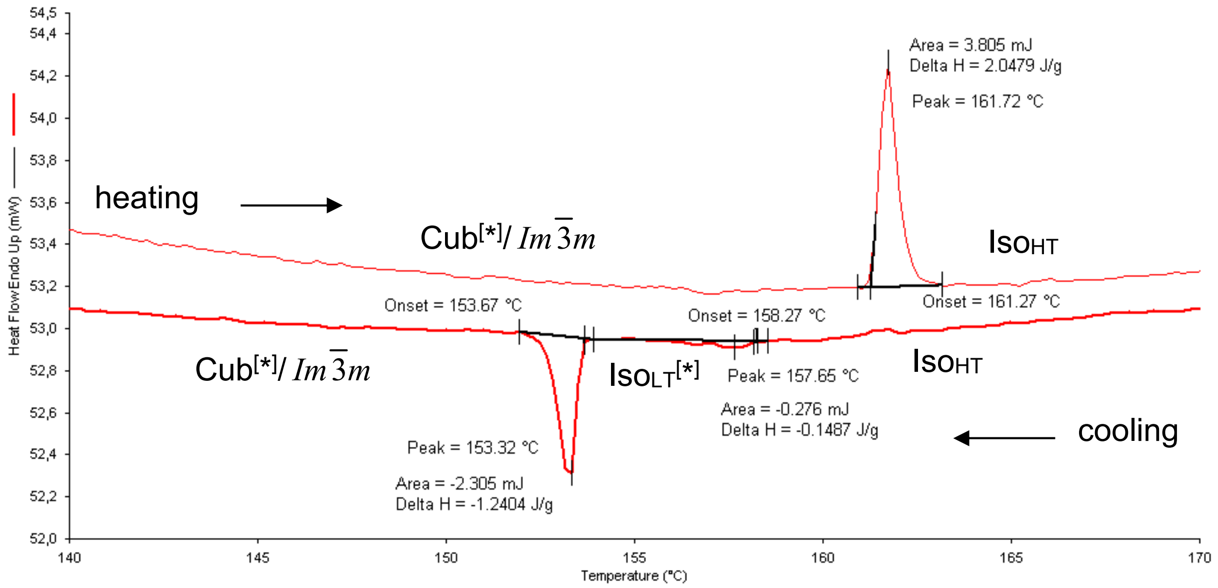


**Figure S9.** Section of the DSC heating and cooling scans (10 K min$^{-1}$) of compound **4** in the temperature range between 140 and 170 °C.

## 3. CD and UV/VIS spectroscopy

UV/VIS spectrum was recorded on Lambda 14 (Perkin-Elmer). Microbeam circular dichroism (CD) spectroscopy experiments were performed at beamline B23 of the Diamond Light Source. An intense synchrotron-generated light beam of a fraction of a mm in diameter was used in the spectrometer, with the ability of samples being scanned in *xy* plane. This allowed only a small number, or even a single domain, to be captured, avoiding signal cancellation through spatial averaging. The beam was deflected vertically through the sample held between two quartz glass windows held in a Linkam hot stage.

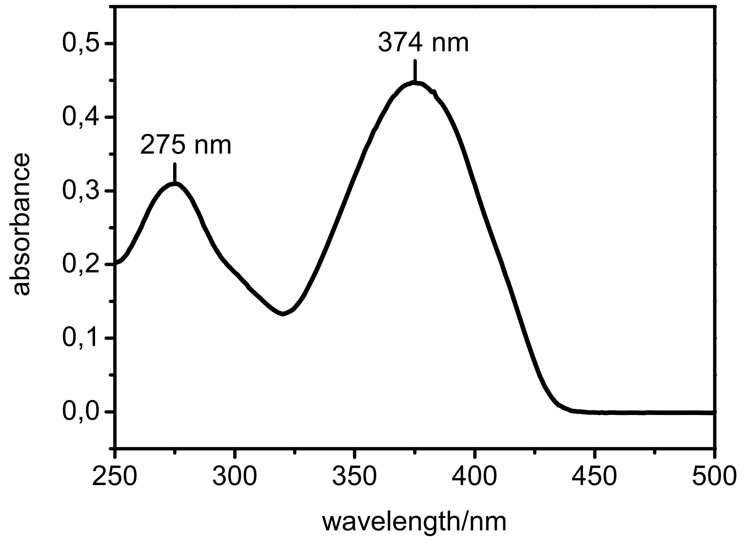


**Figure S10.** UV/VIS spectrum of compound **1g**.

# 4. X-Ray Diffraction

## 4.1 X-ray scattering using laboratory source

X-ray investigations on powder-like samples were carried out with a Guinier film camera (Huber), samples in glass capillaries ($\phi$1 mm) in a temperature-controlled heating stage, quartz-monochromatized $CuK_\alpha$ radiation, 30 to 60 min exposure time, calibration with the powder pattern of $Pb(NO_3)_2$. Aligned samples were obtained on a glass plate. Alignment was achieved upon slow cooling (rate: 1 $K{\cdot}min^{-1}$ – 0.01 $K{\cdot}min^{-1}$) of a small droplet of the sample and takes place at the sample–glass or at the sample–air interface, with domains fiber-like disordered around an axis perpendicular to the interface. The aligned samples were held on a temperature-controlled heating stage and the diffraction patterns were recorded with a 2D detector (HI-STAR, Siemens).

Small- and wide-angle X-ray experiments were also performed using a laboratory beamline based on a Xenocs Genix microfocus source with Fox2d single-bounce curved focusing multilayer optics and aBruker Vantec 2000 multiwire microgap gas detector.

## 4.2 Synchrotron X-ray diffraction and electron density reconstruction

High-resolution small-angle powder diffraction experiments were recorded on Beamline I22 at Diamond Light Source. Samples were held in evacuated 1 mm capillaries. A modified Linkam hot stage with a thermal stability within 0.2 ºC was used, with a hole for the capillary drilled through the silver heating block and mica windows attached to it on each side. A MarCCD detector was used. $q$ calibration and linearization were verified using several orders of layer reflections from silver behemate and a series of $n$-alkanes. The measurement of the positions and intensities of the diffraction peaks is carried out using Galactic PeakSolve$^{TM}$ program, where experimental diffractograms are fitted using Gaussian shaped peaks. The diffraction peaks are indexed on the basis of their peak positions, and the lattice parameters and the space groups are subsequently determined. Once the diffraction intensities are measured and the corresponding space group determined, 3-d electron density maps can be reconstructed, on the basis of the general formula

$$E(xyz) = \Sigma_{hkl} F(hkl) \exp[i2\pi(hx+ky+lz)] \quad \text{(Eqn. 1)}$$

Here $F(hkl)$ is the structure factor of a diffraction peak with index ($hkl$). It is normally a complex number and the experimentally observed diffraction intensity

$$I(hkl) = K\cdot F(hkl)\cdot F^*(hkl) = K\cdot|F(hkl)|^2 \quad \text{(Eqn. 2)}$$

Here K is a constant related to the sample volume, incident beam intensity etc. In this paper we are only interested in the relative electron densities, hence this constant is simply taken to be 1. Thus the electron density

$$E(xyz) = \Sigma_{hkl} \text{sqrt}[I(hkl)] \exp[i2\pi(hx+ky+lz)+\phi_{hkl}] \text{(Eqn. 3)}$$

As the observed diffraction intensity $I(hkl)$ is only related to the amplitude of the structure factor $|F(hkl)|$, the information about the phase of $F(hkl)$, $\phi_{hkl}$, can not be determined directly from experiment. However, the problem is much simplified when the structure of the ordered phase is centrosymmetric, and hence the structure factor $F(hkl)$ is always real and $\phi_{hkl}$ is either 0 or $\pi$.

This makes it possible for a trial-and-error approach, where candidate electron density maps are reconstructed for all possible phase combinations, and the "correct" phase combination is then selected on the merit of the maps, helped by prior physical and chemical knowledge of the system. This is especially useful for the study of nanostructures, where normally only a limited number of diffraction peaks are observed.

Grazing incidence small-angle (GISAXS) experiments were carried out on station BM28 (XMaS line) at European Synchrotron Radiation Facility (ESRF). Thin films were prepared from the melt on a silicon wafer. The thin film coated 5 x 5 $mm^2$ Si plates were placed on top of a custom built heater, which was then mounted on a six-circle goniometer. A MarCCD 165 detector was used. The sample enclosure and the beam pipe were flushed with helium.

### 4.3 Powder diffraction patterns

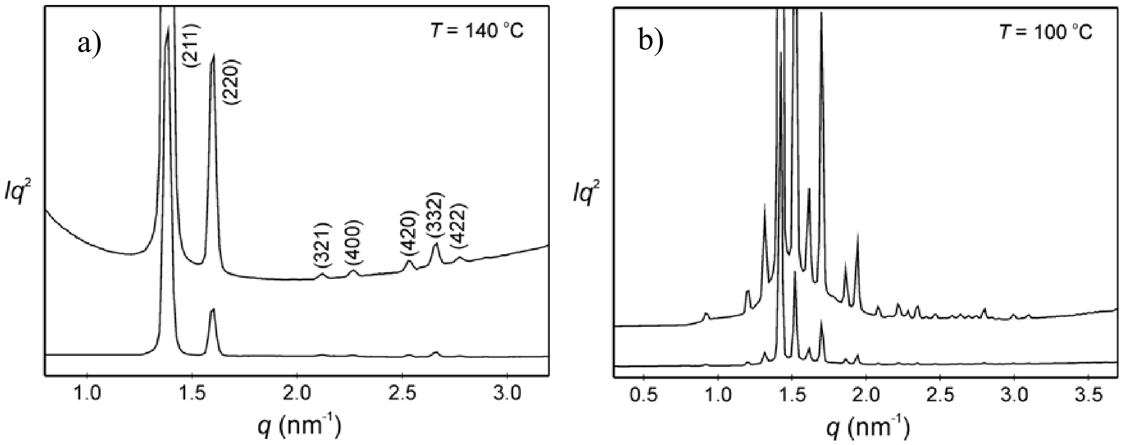


**Figure S11.** Small-angle XRD patterns of (a) the $Ia\overline{3}d$ phase of compound **1c** at 140°C and (b) of the $Im\overline{3}m$ phase of compound **2** at 100 °C, recorded at beamline I22, Diamond Light Source. The upper curves are expanded lower curves.

## 4.4 Tables with *d*-spacings and diffraction intensities

**Table S2.** Experimental and calculated *d*-spacings, relative integrated intensities, and phases used in the reconstruction of electron densities for the Cub/$Ia\bar{3}d$ phase of compound **1a** at 125 ºC. All intensity values are Lorentz and multiplicity corrected.

| (*hkl*) | $d_{obs.}$ – spacing (nm) | $d_{cal.}$ – spacing (nm) | *intensity* | *Phase* |
|---|---|---|---|---|
| (211) | 4.40 | 4.43 | 100 | π |
| (220) | 3.82 | 3.83 | 34.0 | π |
| (222) | 3.13 | 3.13 | 0.24 | 0 |
| (321) | 2.90 | 2.90 | 0.11 | 0 |
| (400) | 2.71 | 2.71 | 1.16 | 0 |
| (420) | 2.43 | 2.42 | 0.44 | 0 |
| (332) | 2.31 | 2.31 | 0.66 | π |
| (422) | 2.21 | 2.21 | 0.05 | π |
| $a_{cub}$= 10.84 nm | | | | |

**Table S3.** Experimental and calculated *d*-spacings, relative integrated intensities, and phases used in the reconstruction of electron densities for the Cub/$Ia\bar{3}d$ phase of compound **1b** at 125 ºC. All intensity values are Lorentz and multiplicity corrected.

| (*hkl*) | $d_{obs.}$ – spacing (nm) | $d_{cal.}$ – spacing (nm) | *intensity* | *phase* |
|---|---|---|---|---|
| (211) | 4.62 | 4.65 | 100 | π |
| (220) | 4.01 | 4.03 | 52.4 | π |
| (321) | 3.04 | 3.04 | 0.14 | 0 |
| (400) | 2.85 | 2.85 | 1.64 | 0 |
| (420) | 2.55 | 2.55 | 0.98 | 0 |
| (332) | 2.43 | 2.43 | 1.09 | π |
| (422) | 2.33 | 2.33 | 0.14 | π |
| $a_{cub}$= 11.40 nm | | | | |

**Table S4.** Experimental and calculated *d*-spacings, relative integrated intensities, and phases used in the reconstruction of electron densities for the Cub/$Ia\bar{3}d$ phase of compound **1c** at 140 ºC. All intensity values are Lorentz and multiplicity corrected.

| (*hkl*) | $d_{obs.}$ – spacing (nm) | $d_{cal.}$ – spacing (nm) | *intensity* | *phase* |
|---|---|---|---|---|
| (211) | 4.55 | 4.55 | 100.0 | π |
| (220) | 3.94 | 3.94 | 25.0 | π |
| (321) | 2.97 | 2.98 | 0.1 | 0 |
| (400) | 2.78 | 2.78 | 1.2 | 0 |
| (420) | 2.48 | 2.49 | 0.5 | 0 |
| (332) | 2.36 | 2.37 | 1.3 | π |
| (422) | 2.27 | 2.27 | 0.3 | π |
| $a_{cub}$= 11.14 nm | | | | |

**Table S5.** Experimental and calculated *d*-spacings, relative integrated intensities, and phases used in the reconstruction of electron densities for the Cub/ $Im\bar{3}m$ phase of compound **1e** at 140 ºC. All intensity values are Lorentz and multiplicity corrected.

| (*hkl*) | $d_{obs.}$ – spacing (nm) | $d_{cal.}$ – spacing (nm) | *intensity* | *phase* |
|---|---|---|---|---|
| (211) | 7.29 | 7.29 | 0.1 | 0 |
| (310) | 5.65 | 5.65 | 0.6 | 0 |
| (222) | 5.16 | 5.16 | 7.7 | 0 |
| (321) | 4.78 | 4.78 | 44.8 | π |
| (400) | 4.47 | 4.47 | 100.0 | 0 |
| (330) | 4.22 | 4.21 | / | / |
| (411) | | 4.21 | 3.2 | π |
| (420) | 4.00 | 4.00 | 10.6 | 0 |
| (422) | 3.65 | 3.65 | 0.9 | π |
| (431) | 3.51 | 3.50 | 1.2 | 0 |
| (510) | | 3.50 | / | / |
| (521) | 3.27 | 3.26 | 0.2 | / |
| (433) | 3.07 | 3.06 | 0.2 | / |
| (530) | | 3.06 | 0.2 | / |
| (442) | 2.98 | 2.98 | 0.1 | / |
| (600) | | 2.98 | 0.4 | / |
| (532) | 2.90 | 2.90 | 0.1 | / |
| (611) | | 2.90 | 0.3 | / |
| (541) | 2.76 | 2.76 | 0.1 | / |
| (631) | 2.64 | 2.64 | 0.03 | / |
| (444) | 2.58 | 2.58 | 0.8 | / |
| (543) | 2.53 | 2.53 | 0.01 | / |
| (550) | | 2.53 | 0.1 | / |
| (710) | | 2.53 | 0.03 | / |
| (640) | 2.48 | 2.48 | 0.2 | / |
| (552) | 2.43 | 2.43 | 0.1 | / |
| (633) | | 2.43 | 0.1 | / |
| (721) | | 2.43 | 0.03 | / |
| (642) | 2.39 | 2.39 | 0.03 | / |
| (651) | 2.27 | 2.27 | 0.03 | / |
| (732) | | 2.27 | 0.03 | / |
| (554) | 2.20 | 2.20 | 0.04 | / |
| (741) | | 2.20 | 0.02 | / |
| (811) | | 2.20 | 0.04 | / |
| $a_{cub}$= 17.87 nm | | | | |

**Table S6.** Experimental and calculated *d*-spacings, relative integrated intensities, and phases used in the reconstruction of electron densities for the Cub/ $Im\bar{3}m$ phase of compound **1f** at 125 ºC. All intensity values are Lorentz and multiplicity corrected.

| (*hkl*) | $d_{obs.}$ – spacing (nm) | $d_{cal.}$ – spacing (nm) | *intensity* | *Phase* |
|---|---|---|---|---|
| (310) | 4.92 | 4.95 | 0.16 | 0 |
| (222) | 4.48 | 4.52 | 1.88 | 0 |
| (321) | 4.16 | 4.19 | 24.22 | π |
| (400) | 3.89 | 3.92 | 100.0 | 0 |
| (330) | 3.68 | 3.69 | / | / |
| (411) | | 3.69 | 0.70 | π |
| (420) | 3.50 | 3.50 | 2.19 | 0 |
| (422) | 3.19 | 3.20 | 0.23 | π |
| (431) | 3.07 | 3.07 | 0.39 | 0 |
| (510) | | 3.07 | / | / |
| (521) | 2.86 | 2.86 | 0.03 | / |
| (433) | 2.69 | 2.69 | 0.04 | / |
| (530) | | 2.69 | 0.04 | / |
| (442) | 2.61 | 2.61 | 0.11 | / |
| (600) | | 2.61 | 0.11 | / |
| (532) | 2.54 | 2.54 | 0.03 | / |
| (611) | | 2.54 | 0.03 | / |
| (541) | 2.42 | 2.42 | 0.02 | / |
| (631) | 2.31 | 2.31 | 0.02 | / |
| (444) | 2.26 | 2.26 | 0.09 | / |
| (543) | 2.22 | 2.21 | 0.01 | / |
| (550) | | 2.21 | 0.01 | / |
| (710) | | 2.21 | 0.01 | / |
| (640) | 2.18 | 2.17 | 0.08 | / |
| (552) | 2.13 | 2.13 | 0.04 | / |
| (633) | | 2.13 | 0.04 | / |
| (721) | | 2.13 | 0.04 | / |
| (642) | 2.10 | 2.09 | 0.02 | / |
| $a_{cub}$= 15.66 nm | | | | |

**Table S7.** Experimental and calculated *d*-spacings, relative integrated intensities, and phases used in the reconstruction of electron densities for the Cub/$Im\bar{3}m$ phase of compound **1g** at 125 ºC. All intensity values are Lorentz and multiplicity corrected.

| (*hkl*) | $d_{obs.}$ – spacing (nm) | $d_{cal.}$ – spacing (nm) | *intensity* | *Phase* |
|---|---|---|---|---|
| (220) | 5.44 | 5.43 | 0.33 | |
| (310) | 4.87 | 4.86 | 0.54 | 0 |
| (222) | 4.44 | 4.43 | 7.00 | 0 |
| (321) | 4.11 | 4.10 | 46.67 | π |
| (400) | 3.84 | 3.84 | 100.0 | 0 |
| (330) | 3.62 | 3.62 | / | / |
| (411) | | 3.62 | 2.75 | π |
| (420) | 3.44 | 3.43 | 5.42 | 0 |
| (422) | 3.13 | 3.13 | 0.50 | π |
| (431) | 3.01 | 3.01 | 0.38 | 0 |
| (510) | | 3.01 | / | / |
| (521) | 2.80 | 2.80 | 0.06 | / |
| (433) | 2.63 | 2.63 | 0.15 | / |
| (530) | | 2.63 | 0.15 | / |
| (442) | 2.56 | 2.56 | 0.13 | / |
| (600) | | 2.56 | 0.13 | / |
| (532) | 2.49 | 2.49 | 0.25 | / |
| (611) | | 2.49 | 0.25 | / |
| (620) | 2.43 | 2.43 | 0.08 | |
| (541) | 2.37 | 2.37 | 0.13 | / |
| (631) | 2.26 | 2.26 | 0.25 | / |
| (444) | 2.22 | 2.22 | 1.50 | / |
| (543) | 2.17 | 2.17 | 0.10 | / |
| (550) | | 2.17 | 0.10 | / |
| (710) | | 2.17 | 0.10 | / |
| (640) | 2.13 | 2.13 | 0.71 | / |
| (552) | 2.09 | 2.09 | 0.63 | / |
| (633) | | 2.09 | 0.63 | / |
| (721) | | 2.09 | 0.63 | / |
| (642) | 2.05 | 2.05 | 0.15 | / |
| $a_{cub}$= 15.36 nm | | | | |

**Table S8.** Experimental and calculated $d$-spacings, relative integrated intensities, and phases used in the reconstruction of electron densities for the Cub/$Im\bar{3}m$ phase of compound **2** at 100 ºC. All intensity values are Lorentz and multiplicity corrected.

| (*hkl*) | $d_{obs.}$ – spacing (nm) | $d_{cal.}$ – spacing (nm) | *intensity* | *phase* |
|---|---|---|---|---|
| (211) | 6.73 | 6.73 | 0.3 | 0 |
| (310) | 5.22 | 5.22 | 0.8 | 0 |
| (222) | 4.76 | 4.76 | 3.1 | 0 |
| (321) | 4.41 | 4.41 | 43.7 | π |
| (400) | 4.13 | 4.12 | 100.0 | 0 |
| (330) | 3.89 | 3.89 | / | / |
| (411) | | 3.89 | 3.6 | π |
| (420) | 3.69 | 3.69 | 10.2 | 0 |
| (422) | 3.37 | 2.37 | 1.2 | π |
| (431) | 3.24 | 3.23 | 1.2 | 0 |
| (510) | | 3.23 | / | / |
| (521) | 3.01 | 3.01 | 0.2 | / |
| (433) | 2.83 | 2.83 | 0.3 | / |
| (530) | | 2.83 | 0.3 | / |
| (442) | 2.75 | 2.75 | 0.1 | / |
| (600) | | 2.75 | 0.4 | / |
| (532) | 2.68 | 2.68 | 0.1 | / |
| (611) | | 2.68 | 0.2 | / |
| (541) | 2.55 | 2.55 | 0.1 | / |
| (631) | 2.42 | 2.43 | 0.1 | / |
| (444) | 2.38 | 2.38 | 0.4 | / |
| (543) | 2.33 | 2.33 | 0.02 | / |
| (550) | | 2.33 | 0.1 | / |
| (710) | | 2.33 | 0.04 | / |
| (640) | 2.29 | 2.29 | 0.1 | / |
| (552) | 2.25 | 2.24 | 0.1 | / |
| (633) | | 2.24 | 0.1 | / |
| (721) | | 2.24 | 0.1 | / |
| (651) | 2.10 | 2.09 | 0.03 | / |
| (732) | | 2.09 | 0.03 | / |
| (554) | 2.03 | 2.03 | 0.1 | / |
| (741) | | 2.03 | 0.03 | / |
| (811) | | 2.03 | 0.1 | / |
| $a_{cub}$= 16.49 nm | | | | |

### 4.5 2D Patterns

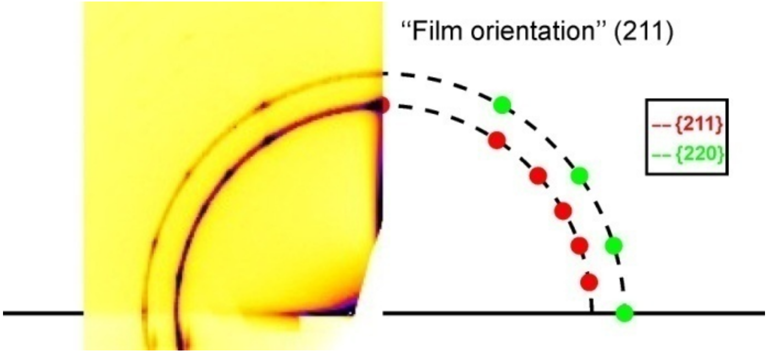


**Figure S12.** Experimental and simulated GISAXS pattern of the $Ia\bar{3}d$ phase of compound **1c**. Spots of different colors belong to different reflection groups (see legend). Simulated spots within a group are generated bypermutation of {*hkl*} indices while keeping one of the {211} planes horizontal.

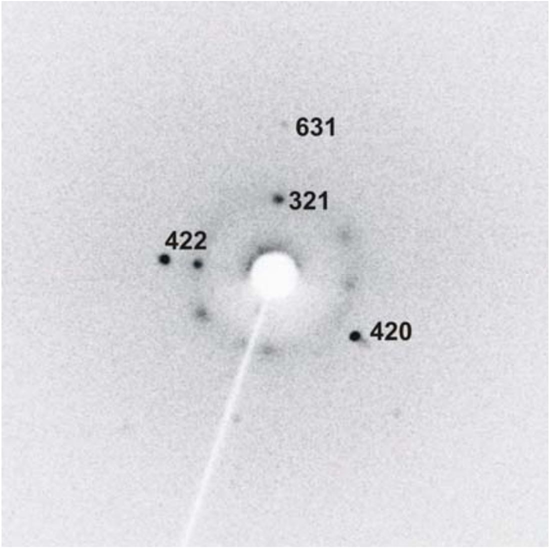


| $2\theta$/° | $\theta$/° | $d_{obs}$/nm | *hkl* | $a/d_{calc}$ | $d_{calc}$/nm | $d_{obs}$-$d_{calc}$/nm |
|---|---|---|---|---|---|---|
| 1.932 | 0.966 | 4.573 | 321 | √12 | 4.541 | 0.03 |
| 2.516 | 1.258 | 3.511 | 420 | √20 | 3.517 | -0.01 |
| 2.764 | 1.382 | 3.196 | 422 | √24 | 3.211 | -0.02 |
| 3.826 | 1.913 | 2.309 | 631 | √46 | 2.319 | -0.01 |
| $a_{cub}$ = 15.73 nm | | | | | | |

**Figure S13**. XRD pattern with indexation of the cubic $Im\bar{3}m$ phase of **3** at $T$ = 140 °C.

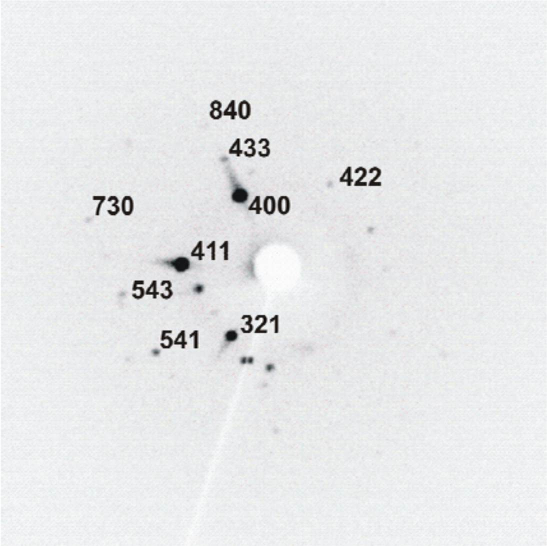


| $2\theta/°$ | $\theta/°$ | $d_{obs}$/nm | *hkl* | $a/d_{calc}$ | $d_{calc}$/nm | $d_{obs}$-$d_{calc}$/nm |
|---|---|---|---|---|---|---|
| 1.932 | 0.966 | 4.573 | 321 | √12 | 4.541 | 0.03 |
| 2.516 | 1.258 | 3.511 | 420 | √20 | 3.517 | -0.01 |
| 2.764 | 1.382 | 3.196 | 422 | √24 | 3.211 | -0.02 |
| 3.826 | 1.913 | 2.309 | 631 | √46 | 2.319 | -0.01 |
| 1.8900 | 0.9450 | 4.674 | 321 | √12 | 4.705 | -0.03 |
| 2.1510 | 1.0755 | 4.107 | 400 | √16 | 4.075 | 0.03 |
| 2.3230 | 1.1615 | 3.803 | 411 | √18 | 3.842 | -0.04 |
| 2.6520 | 1.3260 | 3.331 | 422 | √24 | 3.327 | 0.00 |
| 3.1470 | 1.5735 | 2.807 | 433 | √34 | 2.795 | 0.01 |
| $a_{cub}$ = 16.30 nm | | | | | | |

**Figure S14**. XRD pattern with indexation of the cubic $Im\bar{3}m$ phase of **4** at $T$ = 100 °C.

## 5. Electron density maps

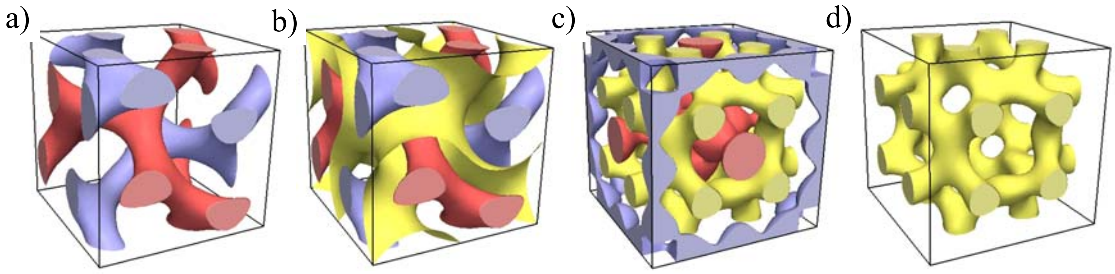


**Figure S15.** Electron density maps of (a, b) the $Ia\bar{3}d$ phase of compound **1c** and (c, d) of the $Im\bar{3}m$ phase of compound **2** reconstructed from the data in the tables above. The isoelectron surfaces enclose the regions of highest electron density, i.e. the aromatic regions. The two

nets are coloured differently, although the density levels are the same. In (b) the minimum surface is also shown in yellow. In (c) the three nets in (a) are coloured differently, although the density levels are the same. The "middle" network is yellow. This network is also shown separately in (d).

# 6. Additional details of structural models

## 6.1 Framework models with minimum surface

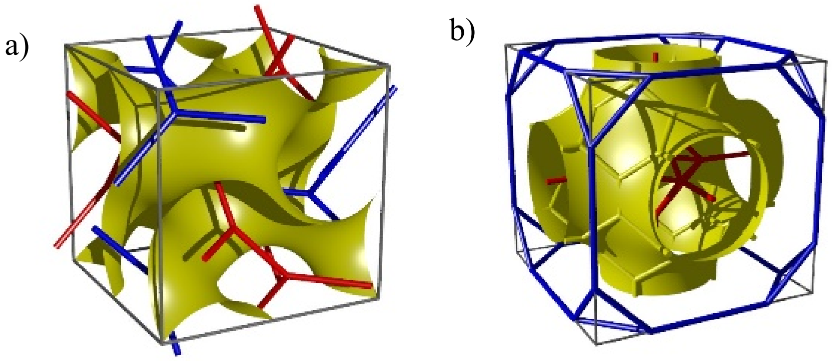


**Figure S16.** Framework models of the $Ia\bar{3}d$ (a) and $Im\bar{3}m$ (b) cubic phases, as in Fig. 1 but with the minimum surface added. In (a) the "gyroid" minimum surface separates the domains of the red and blue infinite networks. In (b) it closely follows the middle (yellow) network.

## 6.2 Calculation of number of molecules and the geometry of cubic networks

The total number of molecules $N$ can be estimated from the lattice parameter $a_{cub}$ (in nm), molar mass $M$(g mol$^{-1}$) and assuming a density of 1.0 g cm$^{-3}$. The equation is

$N = 602.2 \times a_{cub}^3 / M$

### $Im\bar{3}m$ phase

For **1e** ($a_{cub}$ = 17.87 nm and $M$ =1184 g mol$^{-1}$), $N$ is 2.90 x $10^3$

For **1g** ($a_{cub}$ = 15.36 nm and $M$ = 1120 g mol$^{-1}$), $N$ is 1.95 x $10^3$

The middle network contains 24 blue ribbons, 24 red ribbons and 24 green ribbons. The length of the blue ribbion is 0.152$a_{cub}$, and that of the red or green ribbon is 0.203$a_{cub}$, in total the length of the middle network is (0.152 + 2 x 0.203) x 24 x $a_{cub}$ = 13.38 $a_{cub}$. The twisting angle of a blue ribbon is 180° and 240° for red and green ribbons.

The outer and inner networks each contain 12 edges of the octahedron and 3 links between the octahedra, the length of which is 0.325$a_{cub}$ and 0.54$a_{cub}$ respectively. The combined lengths of the outer and inner networks is thus (0.325 x 12 + 0.54 x 3) x 2 x $a_{cub}$ = 11.05 $a_{cub}$.

The total length of all the segments in the unit cell is thus 24.43 $a_{cub}$. Assuming the distance between molecules along the segment is 0.45 nm, the number of "molecular layers" or strata

is 54.29 $a_{cub}$. For **1e** this is 970 layers, for **1g** it is 834 layers. The number of molecules per stratum is thus 3.0 for **1e** and 2.3 for **1g**. The twist angle between successive strata is 180/[(0.152$a_{cub}$)/0.45] = 533/$a_{cub}$ (degrees), which is 30° for **1e** and 35° for **1g**.

### $Ia\bar{3}d$ phase

There are a total of 24 segments in the $Ia\bar{3}d$ unit cell, the length of each segment being 0.354$a_{cub}$. The total twist angle of each segment is 70.5°. The twist angle between successive molecular strata is then 70.5/[(0.354$a_{cub}$)/0.45] = 89.6/$a_{cub}$, which for **1b** ($a_{cub}$ = 11.40 nm) gives 7.9° and for **1a** ($a_{cub}$ = 10.84 nm) is 8.3°.

The total number of molecules per cell can be estimated as 844 for **1b**, and 747 for **1a**. The number of molecules in each segment is thus 35 and 31 respectively. As each segment contains 0.352/0.45 = 0.787$a_{cub}$ molecule strata, the number of molecules per stratum is 3.9 (**1b**) and 3.6 (**1a**).

## 7. Optical microscopy

Polarized optical microscopy experiments were carried out on a Leica DMR XP in conjunction with a heating stage (FP 82 HT, Mettler) and controller (FP 90, Mettler).

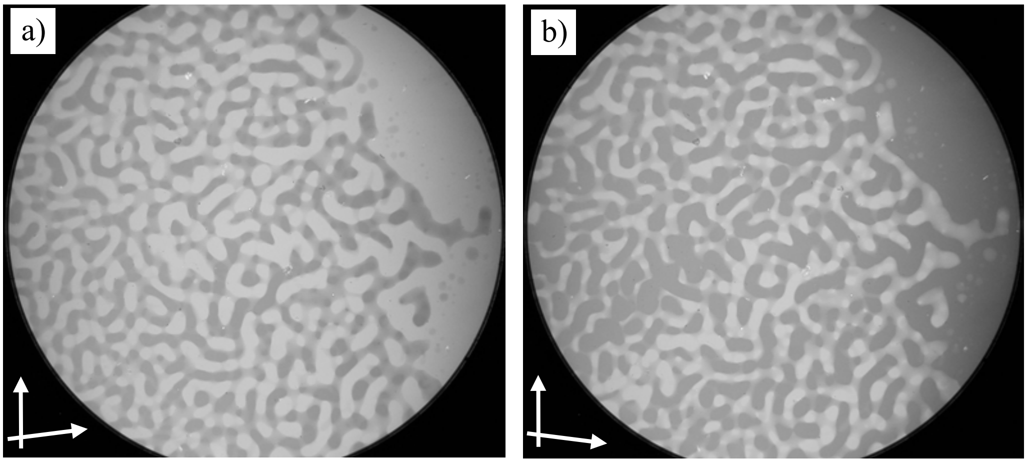


**Figure S17.** Textures with chiral domains of compound **2** in the Cub[*]/$Im\bar{3}m$ phase as observed between slightly decrossed polarizers (±5°) after cooling from the $Iso_{LT}$[*] phase at $T$ = 110 °C.

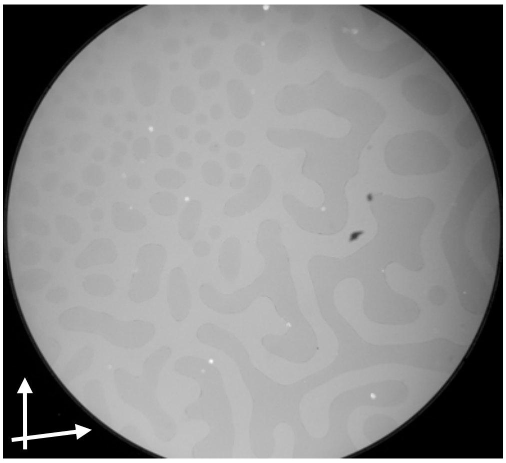

**Figure S18.** Textures with chiral domains in the Cub[*]/ $Im\overline{3}m$ phase of compound **3** as observed between slightly decrossed polarizers after cooling from the $Iso_{LT}$[*] phase at $T$ = 100 °C (b).

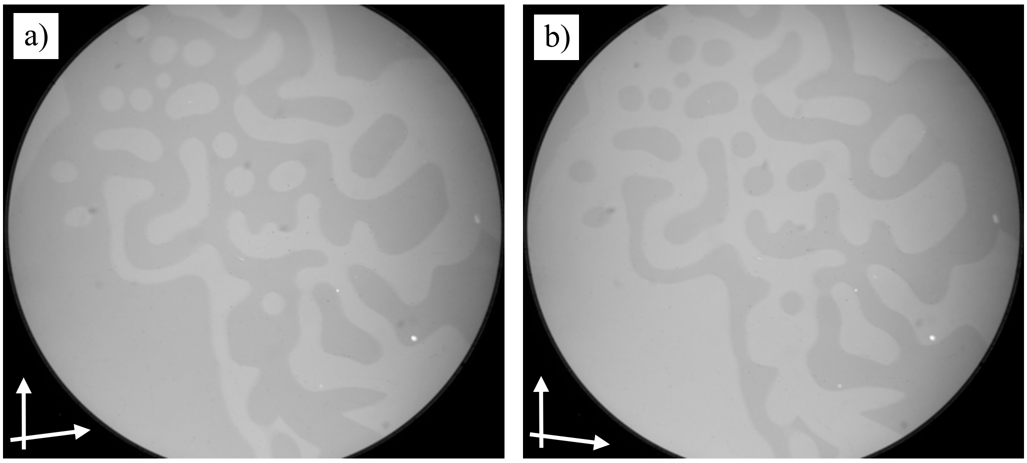


**Figure S19.** Textures with chiral domains in the Cub[*]/ $Im\overline{3}m$ phase of compound **4** as observed between slightly decrossed polarizers (±5°) after cooling from the $Iso_{LT}$[*] phase at $T$ = 145 °C.

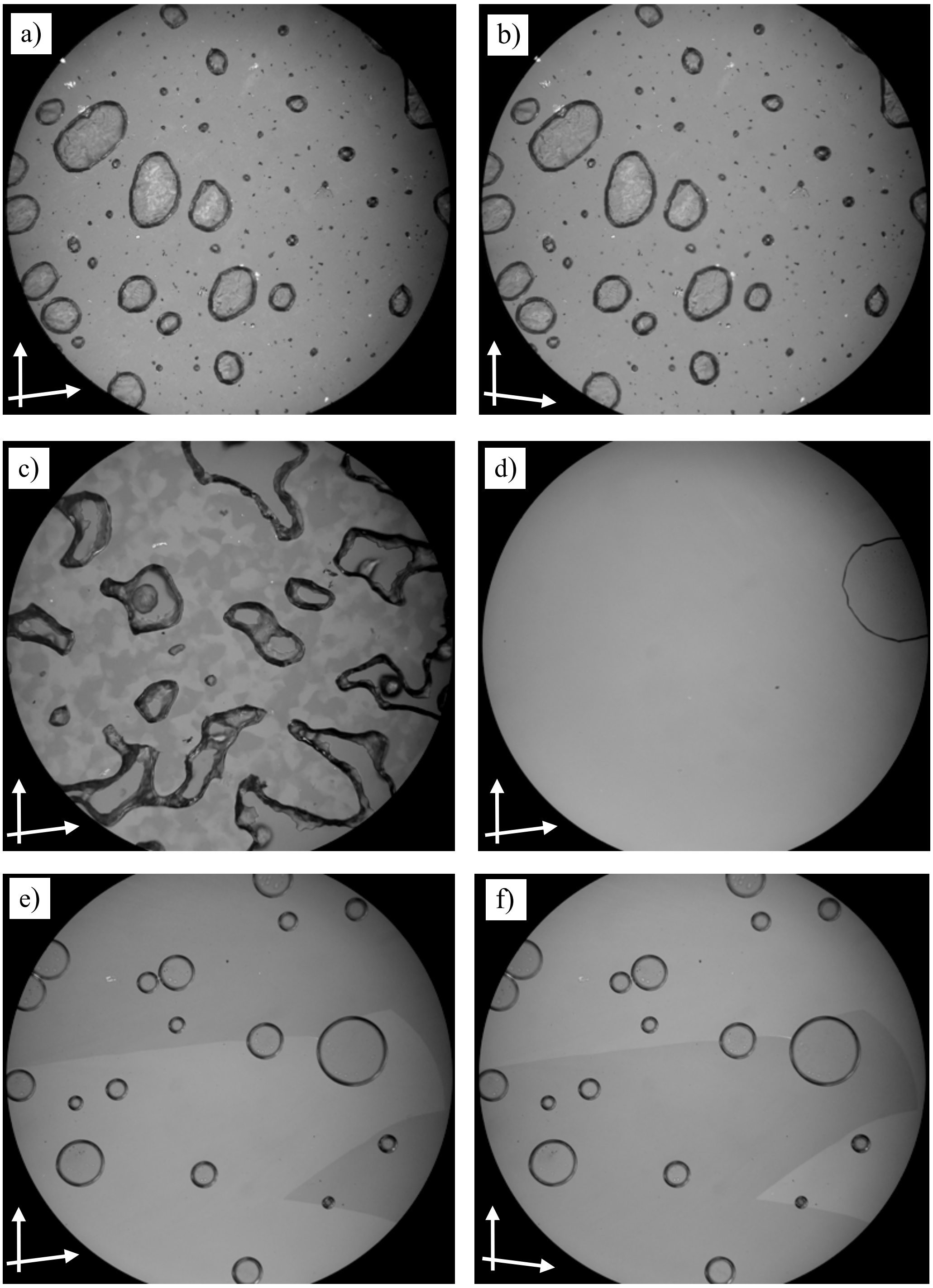


**Figure S20.** Photomicrographs of the cubic phases of compounds **5** (ANBC-*n*) between slightly uncrossed polarizers (±5°). (a, b) Optically inactive $Ia\bar{3}d$ phase of **5a** (ANBC-16) during first heating at $T$ = 180 °C; (c, d) compound **5b** (ANBC-18), (c) chiral $Im\bar{3}m$ phase on first heating at $T$ = 190 °C; (d) optically inactive $Ia\bar{3}d$ phase on cooling from the $Iso_{LT}$ phase at $T$ = 180 °C; (e, f) chiral $Im\bar{3}m$ phase of **5c** (ANBC-20) on cooling from the achiral $Iso_{LT}$ phase at $T$ = 180 °C; the dark lines in a-c) result from poor wetting of glass at the interfaces to the air inclusions after heating the crystalline sample; air bubbles are also present in d-f), this also applies to Fig. S21.

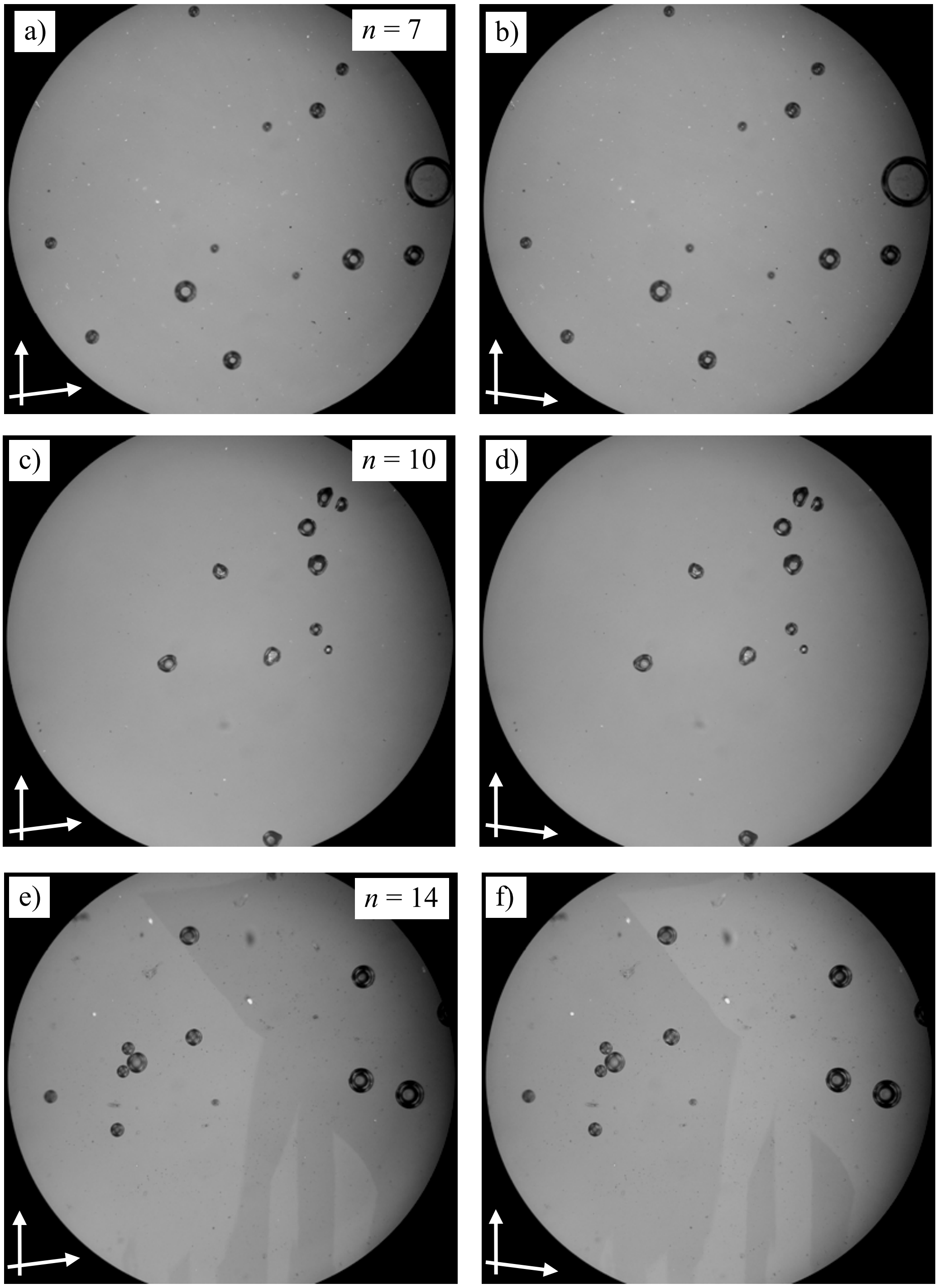


**Figure S21.** Photomicrographs of the cubic phase of different compounds **6** (BABH-*n*) between slightly uncrossed polarizers (±5°) on cooling. (a, b) optically inactive $Ia\bar{3}d$ phase of **6a (**BABH-7) at $T$ = 155 °C; (c), (d) optically inactive $Ia\bar{3}d$ phase of **6b** (BABH-10) at $T$ = 150 °C; (e), (f) Chiral $Im\bar{3}m$ phase of **6c** (BABH-14) at $T$ = 140 °C.

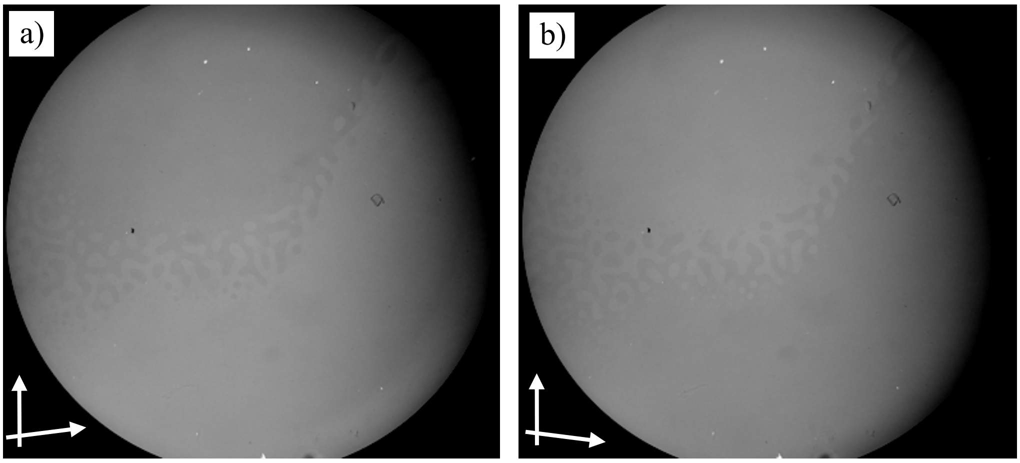


**Figure S22.** Textures with chiral domains of compound **7** as observed between slightly decrossed polarizers (±5°) after cooling from the isotropic at *T* = 55 °C; round boundaries of the chiral domains indicate the existence of an $Iso_{LT}$[*] phase (not reported in [8]).

### 8. Complete references with more than 10 authors in the main text

[8]: L. E. Hough, M. Spannuth, M. Nakata, D. A. Coleman, C. D. Jones, G. Dantlgraber, C. Tschierske, J. Watanabe, E. Körblova, D. M. Walba, J. E. Maclennan, M. A. Glaser, N. A. Clark, *Science* **2009**, *325*, 452;

[12] V. Borshch, Y.-K. Kim, J. Xiang, M. Gao, A. Jakli, V.P. Panov, J.K. Vij, C.T. Imrie, M.G. Tamba, G.H. Mehl, O.D. Lavrentovich, *Nat. Commun.* **2013**, *4*, 2635, DOI: 10.1038/ncomms3635

[13] D. Chen, J. H. Porad, J. B. Hooper, A. Klittnick, Y. Shen, M. R. Tuchband, E. Körblova, D. Bedrovc, D. M. Walba, M. A. Glaser, J. E. Maclennan, N. A. Clark, *Proc. Natl. Acad. Sci. USA* **2013**, *110*, 15931–15936.

## 9. References

S1 Wu, R., Schumm, J. S., Pearson, D. L., Tour, J. M. *J. Org. Chem.* **61**, 6906-6921 (1996).

S2 Yasuda, T. *et al.*. *Adv. Funct. Mater.* **19**, 411-419 (2009).

S3 Miyaura, N., Suzuki, *Chem. Rev.* **95**, 2457-2483 (1995).

S4 Matraszek, J., Mieczkowski, J., Pociecha, D., Gorecka, E., Donnio, B., Guillion, D.. *Chem. Eur. J.* **13**, 3377-3385 (2007).

S5 Kutsumizu, S., Morita, K., Yano, S. *Liq. Cryst.* **29**, 1459-1468 (2002).

S6 Kutsumizu, S., Yamada, M., Yano, S. *Liq. Cryst.* **16**, 1109-1113 (1994).

S7 Kutsumizu, S., Mori, H., Fukatami, M., Naito, S., Sakajiri, K. and Saito, K. *Chem. Mater.* **2008,** *20,* 3675-3687.

S8 Zeng, X., Cseh, L., Mehl, G. H. and Ungar, G. *J. Mater. Chem.* **18**, 2953-2961 (2008).
